# Identification of potential edible mushroom as SARS-CoV-2 main protease inhibitor using rational drug designing approach

**DOI:** 10.1038/s41598-022-05349-x

**Published:** 2022-01-27

**Authors:** Debanjan Sen, Bimal Debnath, Pradip Debnath, Sudhan Debnath, Magdi E. A. Zaki, Vijay H. Masand

**Affiliations:** 1 BCDA College of Pharmacy & Technology, Jessore Road South, Hridaypur, Kolkata, West Bengal 700127 India; 2 Department of Forestry and Biodiversity, Tripura University, Suryamaninagar, Tripura 799022 India; 3 Department of Chemistry, Majaraja Bir Bikram College, Agartala, Tripura 799004 India; 4 Department of Chemistry, Netaji Subhash Mahavidyalaya, Udaipur, Tripura 799114 India; 5 Department of Chemistry, Faculty of Science, Imam Mohammad Ibn Saud Islamic University, Riyadh, 13318 Saudi Arabia; 6 Department of Chemistry, Vidya Bharati Mahavidyalaya, Amravati, Maharashtra 444 602 India

**Keywords:** Virtual drug screening, Computational biology and bioinformatics, Drug discovery

## Abstract

Severe Acute Respiratory Syndrome Coronavirus-2 (SARS-CoV-2) is highly pathogenic to humans and has created health care threats worldwide. This urgent situation has focused the researchers worldwide towards the development of novel vaccine or small molecule therapeutics for SARS-CoV-2. Although several vaccines have already been discovered and are in use for the masses, no therapeutic medication has yet been approved by FDA for the treatment of COVID-19. Keeping this in view, in the present study, we have identified promising hits against the main protease (M^pro^) of SARS-CoV-2 from edible mushrooms. Structure-based virtual screening (VS) of 2433 compounds derived from mushrooms was performed with M^pro^ protein (6LU7). Four promising hits, namely, Kynapcin-12 (M_78), Kynapcin-28 (M_82), Kynapcin-24 (M_83), and Neonambiterphenyls-A (M_366) were identified based on the result of docking, Lipinski’s rule, 100 ns molecular dynamics (MD) simulation and MM/PBSA binding free energy calculations. Finally, the inhibitory properties of these hits were compared with three known inhibitors, baicalein (**1**), baicalin (**2**), and biflavonoid (**3**). Data indicated that M_78, M_82 and M_83 compounds present in edible mushroom *Polyozellus multiplex* were potent inhibitors of M^pro^protein (6LU7). It could be concluded that edible mushroom *Polyozellus multiplex* has potential activity against SARS-CoV-2 infection and identified molecules could be further explored as therapeutic inhibitors against SARS-CoV-2.

## Introduction

Pandemic COVID-19 caused by SARS-CoV-2 virus has posed serious challenges to the research community, health workers, and government officials worldwide. Due to the rapid human-to-human contagious nature of SARS-CoV-2, the disease adversely affected 241.2537 million people, with 4.9116 million fatalities in 223 countries and territories around the world (report as of 17th October 2021; https://www.worldometers.info/coronavirus/). The disease drastically hit the economic growth worldwide and pushed millions of people towards unemployment. The SARS-CoV-2 was initially detected at the end of December 2019, in Wuhan City, characterized by an atypical pneumonia outbreak^1–3^. The World Health Organization (WHO) declared COVID-19 as a global public health emergency of international concern and in March 2020, it was declared as a pandemic. Under this emergency, scientists all over the world have been working for the development of novel vaccines and drug molecules to prevent and treat the COVID-19 disease. Recently, several manufacturers such as Pfizer Biotech, AstraZeneca University of Oxford, Serum Institute of India Pvt. Ltd, Moderna Biotech, Sinopharm / BIBP have launched vaccines in the market to combat COVID-19. Unfortunately, no therapeutic medication has yet been approved by FDA for the treatment of this disease.

The human coronaviruses genome has several conserved structural proteins such as Spike (S) glycoprotein, envelope (E) protein, membrane (M) protein, and nucleocapsid (N) protein. It has at least four non-structural proteins (nsPs) such as- 3-chymotrypsin-like protease (3CL^pro^) also known as Main protease (M^pro^), papain-like protease (PL^pro^), helicase, and RNA-dependent RNA polymerase (RdRp)^4^. Protein sequence alignment analyses of SARS-CoV-2 indicated that catalytic sites of the four SARS-CoV-2 enzymes which could serve as antiviral targets are highly conserved and show a total of 79.9% genomic similarity with SARS-CoV^5^. This attribute could be utilized to understand and inhibit the replication cycle of SARS-CoV-2. The non-structural proteins (nsPs), 3CL^pro^ and PL^pro^, which are two important proteases, play a crucial role in the viral replication process through the extensive proteolysis of two replicase polyproteins, pp1a and pp1ab into 16 non-structural proteins (nsP1-nsP16)^6^. These nsPs are assembled and form the replication-transcription complex which regulates various functions of virus replication viz. replication of the viral genome, sub-genomic RNA processing, and packaging of the new virion^7^. Interrupting any replication process would become a potential molecular target to develop therapeutics against coronavirus.

The urgent need for drugs to treat COVID-19 has led scientists to focus on protease inhibitors as potential drugs for the treatment of COVID-19 patients. In this regard, M^pro^ has been found to be highly sensitive, therefore, it has been considered as a key therapeutic target for the development of a drug against coronavirus^8,9^. As a treatment strategy against COVID-19, a combination of anti-HIV protease drugs, lopinavir and ritonavir, was currently employed to treat the COVID-19 patients with mild and moderate infections^10,11^. However, the patients’ outcome treated with this combination suggested that the curative effect of these drugs is minimal with potentially toxic side effects that might be harmful to the patients^12^. Some other repurposed drugs are also currently used, taking the advantage of drug safety, to treat the COVID-19 patients as a short-term and non-specific solution^13–16^. Identifying bioactive compounds from the natural sources, which could inhibit SARS-CoV-2 main protease, has been considered as an alternative approach to combat COVID-19. In silico techniques provide promising preliminary evidence for drug discovery in a shorter span of period. Recently, several researchers have focused on identifying potential biomolecules active against SARS-CoV-2 from natural sources by implication of in silico drugs designing approach^17–21^. This is because phytochemicals have been used as a good source of antiviral drugs in folk medicine to treat viral infections. Moreover, drug molecules identified from natural resources, especially plants have minimal side effects associated with them. Therefore, the development of more targeted inhibitors from natural sources could be an efficient therapeutic strategy to combat COVID-19.

Mushrooms are rich in low-calorie fibre, protein, health-boosting vitamins, and minerals. It is used as food due to its great taste and amazing health benefits worldwide. Mushrooms raised with exposure to ultraviolet light are a good source of Vitamin D^22^. There is a common belief that supplementation of mushrooms in dietary meals reduces the health care expenditure and remove the fear of the influenza outbreak^23^. Recent pharmacological studies indicated that mushrooms are an exceptional source of several bioactive molecules, possessing antiviral^24,25^, anti-inflammatory^26,27^, antioxidants, antifungal, anticancer, antibacterial, and inhibition of platelet aggregation activities^28^. Mushrooms exhibited strong anti-viral properties when used against influenza-A virus^29–31^, Dengue virus serotype 2^32^, HIV-1, HIV-2^33,34^, type-2 herpes simplex viruses^35^, pandemic H1N1 and human H3N2^36^. Many patients have continually suffered from inflammatory complications due to cytokine storms because of the elevated levels of ILs, IFN-γ, tumour necrosis factor α (TNF-α), interferon gamma-induced protein (IP10), and granulocyte colony-stimulating factor (GCSF)^37^. The major life-threatening event associated with the COVID-19 infection is cytokine storm^37^. More importantly, studies have shown that several edible mushrooms boost up immune responses by stimulating the immune effect or cells like cytotoxic T lymphocytes (TCL), T-cells, dendritic cells (DCs), natural killer cells, and macrophages, which further induced the expression and secretion of cytokines including interleukins (ILs) and interferon-gamma (INF-γ)^38,39^. These exciting medicinal properties of mushrooms have led us to investigate their therapeutic potential against the COVID-19^40^. The main aim of the present study was to identify potential edible mushrooms with compounds having a high binding affinity towards SARS-CoV-2 M^pro^. Recently, Rangsinth et al. have carried out an in-silico study of mushroom compounds against the main protease of SARS-CoV-2^41^. However, they have investigated only 36 compounds that have been reported to possess anti-HIV protease properties. In our present study, we carried out a detailed investigation of 2433 mushroom compounds for their potential as SARS-CoV-2 main protease inhibitors using VS, MD simulation, Lipinski’s rule, MM/PBSA binding free energy calculation, and comparison with known inhibitors. We observed that several phenolic compounds of mushrooms exhibited strong binding affinity with the main protease of SARS-CoV-2. Based on the results obtained, we believe that further in-vitro and in vivo studies of the reported compounds may provide more scientific information about the inhibitory properties of these mushrooms.

## Materials and methods

### Data collection and preparation target protein

In the present study, a databank of 2433 compounds was retrieved from different mushrooms available in the literature^42–47^ and mushroom compounds from the food databank (https://foodb.ca/). Structures of all compounds retrieved from the literature were drawn using ChemDraw Professional 15.1 and saved in the sdf format. After importing all the ligand files in the Maestro version 12.3 used under academic license, a single file was prepared. Then the prepared single-file was imported into the PyRx software tool. The UFF force field^48^ was used to convert all the ligands in the pdbqt format, followed by energy minimization. The X-ray crystal structure of SARS-CoV-2 M^pro^ (PDB ID: 6LU7, resolution: 2.16 Å) was downloaded from the RCSB protein databank (http://www.rcsb.org/)^49,50^. The previously prepared protein pdbqt file of M^pro^^17^ was used for the docking purpose in both AutoDock Vina (ADV) in PyRx and AutoDock 4.2 (AD), both are open-source software. The top fourteen hits resulted from virtual screening were again re-docked using AutoDock 4.2^51^ software considering identical grid parameters.

### Receptor grid generation, RMSD calculation, virtual screening, and molecular docking

The AutoDock Vina integrated with PyRx software^52–54^ was used to perform the virtual screening installed in a Windows 10 Operating System supported by Intel i5 8600 K processor with 8 GB RAM. The grid dimension of the main protease was fixed by selecting active site amino acid residue information (HIS-41, MET-49, PHE-140, LEU-141, GLY-143, CYS-145, HIS-163, GLU-166, GLN-189). The grid centre coordinate of M^pro^ were –10.88, 13.93, 68.21 along the X, Y and Z axis, respectively and grid size were 58, 68, 70 along X, Y and Z axis, respectively with grid spacing 0.375. The energy range was set at 4 and exhaustiveness was set at 8.0. For docking with protonated target, protonation was done by using H +  + server version 3.2 (http://biophysics.cs.vt.edu/H + +)^55^ at pH = 6.5, internal dielectric = 10.0, external dielectric = 80.0 and salinity 0.15. The docking score of coligand (N3) was considered as the standard reference. Validation of the docking protocol is a crucial step before performing docking-based virtual screening. The docking protocol was validated by measuring the root mean square deviation (RMSD) using PyMOL 2.5. The compounds and co-ligand was prepared using the default parameters of PyRx. For calculation of RMSD, each docking poses of N3 generated during the docking program were superimposed on the native conformation of N3, using the “pair_fit” command in PyMOL software (http://www.pymol.org). The output compound and protein in pdbqt format were imported in PyMOL 2.5 for visual inspection of binding poses, followed by the export of the protein–ligand complex in PDB. The protein–ligand complexes imported in ProteinsPlus server (https://proteins.plus)^56,57^ and their 2D interactions were analyzed.

### Drug-likeness properties prediction

Nearly 40% of the identified candidate drugs fail in the clinical trials due to the poor ADME properties^58^. Therefore, prediction of the five physicochemical parameters such as molecular weight, number of H-bond acceptors, number of H-bond donors, molar refractivity, n-octanol/water partition coefficient, i.e., Lipinski’s rule of five^59^ of the selected hits was performed using publicly available online server SwissADME (http://www.swissadme.ch)^60^.

### Molecular dynamic simulation protocol

All-atom molecular dynamics simulation (MDS) of the selected hits was conducted by Gromacs 2018.1^61^ software supported by NVIDIA RTX 2070 GPU and Intel i7 990 k processor running over Linuxmint 19.3 Operating System (OS). The pdb2gmx program of the Gromacs 2018.1 package with Charmm36^62^ force field was used to prepare the protein topology. Topology for each ligand was obtained from the SwissParamTool^63^, an online server-based parameterization program. After rejoining the protein and ligand topology, each system was solvated using TIP3P^64^ water model into a (10Åx10Åx10Å) cubic box. Adequate numbers of Na^+^ and Cl^‒^ ions (0.15 M) were added to neutralize each solvated protein–ligand system. The steepest descent algorithm^65^ was used to minimize each system with a maximum of 50,000 steps, and the force was set to less than10.0 kjole/mol. In the two-stage equilibration step, the 1st step is the NVT ensemble step in which the volume, temperature, and number of particles were kept constant and maintained for 2 ns. The 2nd step is the NPT ensemble step which has constant pressure along with equilibration of temperature and numbers of particles for 10 ns. For each equilibration step, 100 ns positional restraint of C_α_ atoms were applied. Free movement of the solvent molecules was allowed to maintain the solvent equilibrium. The linear constraint solver algorithm^66^ was used to constrain the covalent bonds of the system. The particle mesh Eshwald (PME)^67^ method was applied for long-range electrostatic interaction setting cutoff of 1.2 nm and Fourier spacing of 1.2 nm. The V-rescale weak coupling method^68^ was used to regulate the temperature (310.15 K) of the system. The Parrinello–Rahman method^69^ was used to regulate 1 atm pressure, density, and total energy of the system. Each equilibrated system with acceptable geometry and solvent orientation was subjected to100 ns production run without setting any restraint followed by a 2 fs time step. The structural coordinates were recorded in every 2 ps interval. After the successful completion of the MDS, water and ions were stripped out, followed by PBC correction to refine the trajectories. From the refined trajectories, various parameters like root mean square deviation (RMSD)^70^, root mean square fluctuation (RMSF)^71^, the radius of gyration (Rg)^72^, and solvent accessible surface area (SASA)^73^ that occurred in between ligand and protein were calculated considering co-crystal coordinates as a reference structure. The VMD1.9^74^ program was used to visualize the trajectory and render images. Grace 5.1.25 software (https://plasma-gate.weizmann.ac.il/Grace) was used as a plotting program. The stability of MD complexes was also evaluated using the centre of mass (CoM) distance.

### Molecular mechanics Poisson-Boltzmann surface area (MM/PBSA) calculation protocol

The ligand–protein binding interaction was quantitatively estimated by a widely acceptable Molecular Mechanics Poisson-Boltzmann Surface Area (MM/PBSA) approach^75^. The g_mmpbsa script program^76^, a high throughput MM/PBSA calculation tool in Gromacs software, was used to perform MM/PBSA based binding free energy (∆G_bind_) calculation [https://rashmikumari.github.io/g_mmpbsa/Tutorial.html]. The g_mmpbsa script program, along with the APBS 1.4 program^77^ was used to calculate the above terms. To perform the calculations mentioned above, snapshots of the last 10 ns frames were extracted from the total trajectory using the gmaxtrjconv command. The total 100 ns trajectory frames were supplied as an input for the g_mmpbsa program to calculate the binding energy.

## Results and discussion

### Molecular docking and Lipinski’s rule analysis

The M^pro^ binding site interactions were assigned by importing the protein–ligand crystal structure in the ProteinsPlus server. The detailed active site interacting amino acid residues included LEU-4, GLY-143, HIS-163, GLU-166, and GLN-189. Therefore, a receptor grid box was prepared by selecting these active site amino acid residues. The RMSD value between the co-ligand and docking pose of the same was 1.47Å^17^. This value was less than 2.0 Å; therefore, the validation of the docking protocol was regarded to be successful. This indicates that there is little visible difference between the docked pose of colignd to the original crystallographic bound ligand pose. In the first step, databases containing 2433 numbers of compounds derived from mushrooms were docked with a ligand-free M^pro^ active site using AutoDock Vina in PyRx. Amongst the top-scored hits extracted from the docking, fourteen hits with docking scores ≤ − 7.0 kcal/mol were subjected to AD to eliminate the false positives. Lower the value of docking score (i.e. higher negative value) of a ligand indicates a higher binding affinity towards the target protein. Out of eight docked conformations generated during the docking with ADV of each ligand, their highest binding energy conformation was selected for 2D visualization of interactions (Fig. S1). In the crystal structure of SARS-CoV-2 M^pro^ (PDB ID: 6LU7), the co-ligand (N3) of SARS-CoV-2 M^pro^ was covalently bonded with CYS-145. This co-ligand was isolated from the protein–ligand crystal structure and re-docked into the active site of M^pro^. The docking score of co-ligand was − 7.2 kcal/mol and this value was used as a control value to reduce the chemical space after docking. The hits with the docking score of ≤ − 7.2 were selected as SARS-CoV-2 M^pro^ inhibitors, source mushroom of the selected hits and trivial name of the hits have been summarized in Table 1. The docking score predicted by the ADV of top ten hits ≤ − 8.0, are M_78, M_82, M_83, M_88, M_111, M_112, M_201, M_366, M_421 and M_505. The structural insights into the binding interaction of these hits with SARS-COV-2 M^pro^ were analyzed using ProteinsPlus are shown in Fig. S1. Zhang et al. showed that α-ketoamide inhibitor bound with the active site of M^pro^, involving, HIS-41, HIS-164 and CYS-145 amino acid residues^78^. Yoshino et al. also performed long-time molecular dynamics (MD) simulation of M^pro^ with three drug-like peptide candidates and identified the crucial active site amino acid residues HIS-41, GLY-143, and GLU-166 involved in the inhibition of M^pro^^79^. Along with docking score, interaction with crucial amino acid residues may be other important criteria in the selection of potential inhibitors. The ADV docking score of ten hits was less than the ADV docking score of known inhibitors **1** and was comparable to other known inhibitors **2** and **3** (Table S1). The hits M_78, M_82, M_83, and M_201 interacted with the two crucial amino acid residues viz. HIS-41, and GLU-166 along with the other interacting residues. The hit M_111 interacted with the three crucial amino acid residues viz. HIS-41, CYS-145, GLU-164, along with five more interactions. Along with the other interacting amino acid residues, the hits M_88 and M_112 interacted with the GLY-143, GLU-166, and CYS-145, GLU-166, respectively. The other hits M_366, M_421, and M_505 interacted with the crucial amino acid residues, HIS-41 or GLU-166. All the top hits also showed the AD docking score less than AD docking score of two known inhibitors **1** and **2** (Table 1). The docking results of the fourteen selected hits with protonated M^pro^ (Fig. S2) were very close to the docking score of hits with nonprotonated M^pro^. The docking score with protonated M^pro^ have been shown in Table S2. Further, all the ten top hits were deeply inserted into the active site of M^pro^. The 3D surface topology pose of hits M_78, M_82, M_83, M_88, M_111, M_112, M_201, M_366, M_421 and M_505 in the active site have been shown in Fig. S3. The binding pose of inhibitors M_78, M_82, M_83, M_88, M_111, M_112, and M_336 superimposed on the binding pose of known inhibitors **1** and **2**. It was observed that the pharmacophoe of most of the selected top-scored hits matched with the known inhibitors. A visual inspection of selected inhibitors superimposed on known inhibitors is shown in Fig. S4.

**Table 1:** Source mushroom of the **c**ompounds, the structure of the top fourteen hits, trivial name of the compounds, docking score (*ADV = Auto Dock Vina, ^**^BSI = Binding Site Interaction, ^#^AD = Auto Dock 4.2), interacting active site residues.

Sl no	Compound source	Structure of the selected hits and trivial name	*ADV score kcal/mol	**BSI	^#^AD score kcal/mol	Ki
1	M_01 *Ganoderma pfeifferin* Bres Family: Ganodermataceae		− 7.5	H-bonding: ASN-142, HIS-163, THR-190; Hydropbobic: MET-165, GLN-189, ARG-188	− 6.47	18.02 μM
2	M_60 *Lepista sordida* (Schumach.), Edible mushroom, Family: Tricholomataceae		− 7.0	H-bonding: GLU-166, ARG-188 Hydropbobic: MET-165, GLN-189	− 7.38;	3.87 μM
3	M_62 *Lepista sordida* (Schumach.) Edible mushroom, Family: Tricholomataceae		− 7.0	H-bonding: GLU-166, ARG-188 Hydropbobic: MET-165, GLN-189	− 7.37	3.97 μM
4	M_77 *Polyozellus multiplex* (Underw.) Murrill Korean edible mushroom, Family: Thelephoraceae		− 7.4	H-bonding: GLU-166, ASP-187; Hydropbobic: MET-49, MET-165	− 8.03	1. 29 μM
5	M_78 *Polyozellus multiplex* (Underw.) Murrill., Korean edible mushroom, Family: Thelephoraceae		− 8.1	H-bonding: PHE-140, GLU-166, ASP-187; Hydropbobic: HIS-41, GLU-166	− 7.77	2.02 μM
6	M_82 *Polyozellus multiplex* (Underw.) Murrill Korean edible mushroom, Family: Thelephoraceae		− 8.5	H-bonding: LEU-141, SER-144, GLU-166, ASP-187; Hydropbobic: HIS-41, MET-165	− 7.54	3.72 μM
7	M_83 *Polyozellus multiplex* (Underw.) Murrill Korean edible mushroom, Family: Thelephoraceae		− 8.3	H-bonding: LEU-141, SER-144, GLU-166, ASP-87; Hydropbobic: HIS-41, MET-165	− 8.58	512.25 nM
8	M_88 *Sarcodonim bricatus* (L.) P. Karst, Edible mushroom, Family: Bankeraceae		− 8.0	H-bonding: GLU-166, ASP-187, GLY-143	− 9.08	219.43 nM
9	M_111 *Thelephora ganbajun* M. Zang, Edible mushroom, Family:Thelephoraceae		− 8.8	H-bonding: PHE-140, CYS-145, HIS-164, ASP-187; Hydropbobic: HIS-41, MET-49, MET-165, GLN-189	− 10.2	33.64 nM
10	M_112 *Thelephora ganbajun* M. Zang, Edible mushroom, Family:Thelephoraceae		− 8.0	H-bonding: PHE-140, CYS-145, GLU-166, ASP-187;	− 7.52	3.85 μM
11	M_201 *Neonothopanus nambi* (bioluminescent Mushroom)		-8.5	H-bonding: CYS-44, GLU-166 Hydrophobic: HIS-41, MET-165, ARG-188	-8.00	1.21 μM
12	M_366 *Neonothopanus nambi* (bioluminescent Mushroom)		− 8.3	H-bonding: CYS-44 Hydrophobic: HIS-41, MET-165, ARG-188	− 8.73	401.89 nM
13	M_421 *Neonothopanus nambi* (bioluminescent Mushroom)		− 9.3	H-bonding: GLU-166	− 8.45	638.15 nM
14	M_505 *Neonothopanus nambi* (bioluminescent Mushroom)		− 8.8	H-bonding: HIS-41	− 8.92	290.54 nM
15	Co-ligand N3		− 7.2	H-bonding: ALA-2, GLY-143, HIS-163, GLU-166; Hydrophobic: LEU-4, MET-49, GLN-189	− 6.63	13.71 μM

**Figure 1:**
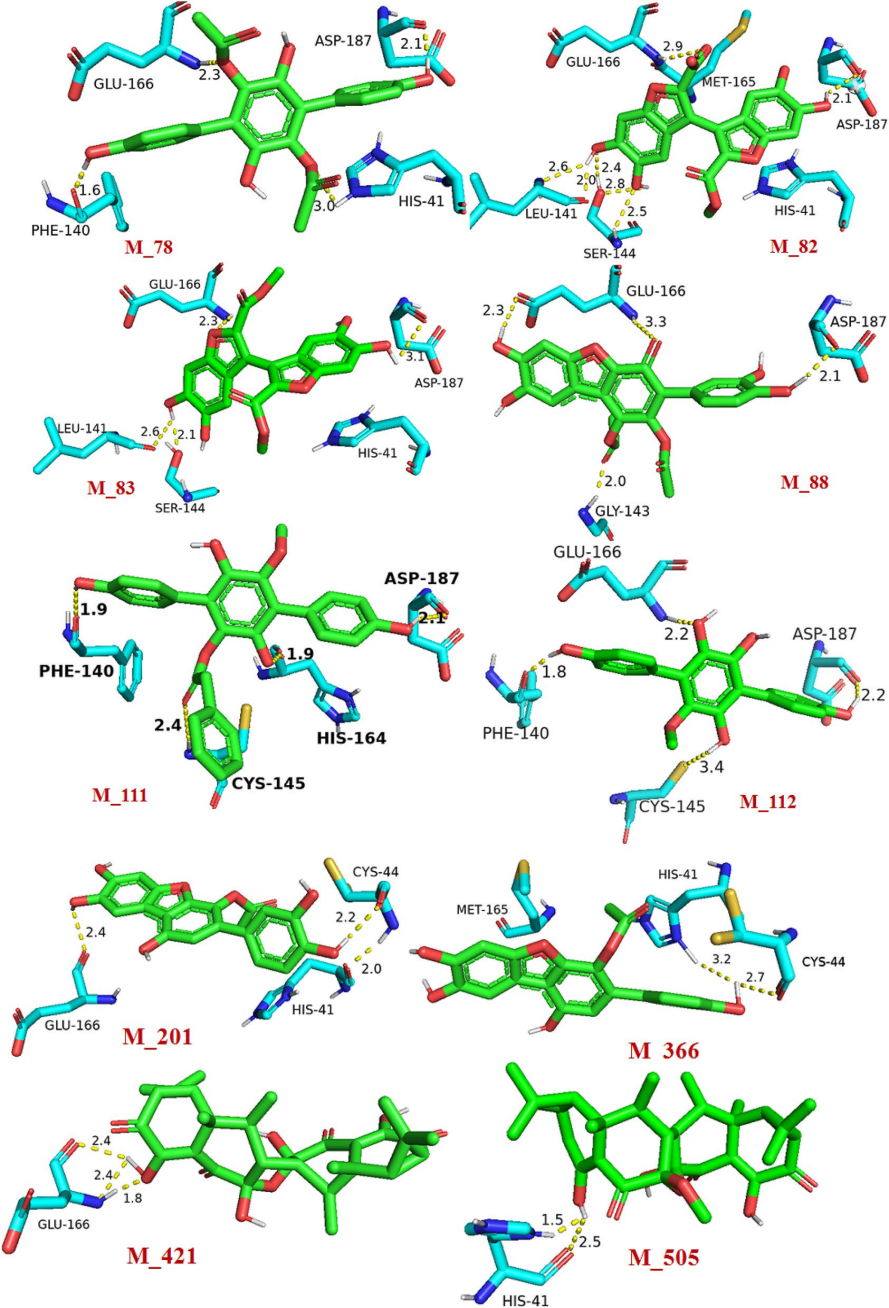
Detailed view of 3D interactions of selected hits (green stick) with SARS-CoV-2 M^pro^ active site amino acid residues (cyan stick) and their interacting distances. The hydrogen-bonding interactions were depicted in a yellow dotted line.

Most of the top ten hit exhibited close (< 3.0 Å) hydrogen bond donor and hydrogen bond acceptor interactions. These short-distance interactions revealed the efficient binding affinity of the hits towards M^pro^. The detailed view of the 3D interaction often hits with SARS-CoV-2 M^pro^ active site amino acid residues have been depicted in Fig. 1. The molecular weights of all the top hits were in the range of 340.33‒540.60. The acceptable range of molecular weight is ≤ 500. Therefore, the molecular weight of all the hits, except M_421 and M_505 were in the acceptable range. The number of hydrogen bond acceptors of all the hits was also in the range of 6‒10. The acceptable range of this is ≤ 10. Therefore, the numbers of hydrogen bond acceptor values of all the selected hits were in the acceptable range. The range of hydrogen bond donor groups of all the selected hits was 4‒5, and the acceptable range is ≤ 5; this property was also in the acceptable range.

For a drug-like molecule, the molar refractivity should be between 40 and 130, and here all the hits were in this range except M_421 and M_505. The *n*-octanol/water partition coefficient of all the hits should be ≤ 5; this value for all the selected hits was in the acceptable range in the present study. Therefore, the Lipinski rule of five dealing with 90% of the orally active drugs that have achieved phase II clinical status was obeyed by the hits M_78, M_82, M_83, M_88, M_111, M_112, M_201, M_366. Among the mushroom compounds based on docking score and interaction with amino acid residues, and drug-like charactersM_78, M_82, M_83, M_88, M_111, M_112, M_201, M_366 were selected for the MD simulation study. The physiochemical parameters of the top ten proposed inhibitors have been shown in Table 2.

**Table 2:** Physiochemical parameters of selected SARS-CoV-2 M^pro^ proposed inhibitors from mushrooms.

Parameters	M_78	M_82	M_83	M_88	M_111	M_112	M_201	M_366	M_421	M_505
MW	410.37	399.28	414.32	439.35	458.46	340.33	382.32	366.32	540.60	554.63
Acceptable Range	≤ 500	≤ 500	≤ 500	≤ 500	≤ 500	≤ 500	≤ 500	≤ 500	≤ 500	≤ 500
NHBA	8	10	10	10	7	6	8	7	9	9
Acceptable Range	≤ 10	≤ 10	≤ 10	≤ 10	≤ 10	≤ 10	≤ 10	≤ 10	≤ 10	≤ 10
NHBD	4	4	4	4	4	5	5	4	4	3
Acceptable Range	≤ 5	≤ 5	≤ 5	≤ 5	≤ 5	≤ 5	≤ 5	≤ 5	≤ 5	≤ 5
MR	108.40	95.81	102.07	110.38	127.88	93.92	100.77	98.75	136.97	141.70
Acceptable Range	40–130	40–130	40–130	40–130	40–130	40–130	40–130	40–130	40–130	40–130
iLOGp	2.39	1.48	2.37	2.26	2.72	2.15	1.74	1.83	3.811	2.93
Acceptable Range	≤ 5	≤ 5	≤ 5	≤ 5	≤ 5	≤ 5	≤ 5	≤ 5	≤ 5	≤ 5

The parameters *MW* molecular weight, *NHBA* number of H-bond acceptors, *NHBA* number of H-bond donors, MR molar refractivity, *iLOGp* n-Octanol/Water partition coefficient).

### Molecular dynamics simulation properties analysis

The application of the molecular dynamics simulation is a widely accepted approach for predicting the protein–ligand complex's stability. The 100 ns atomistic MD simulation was performed to explore the dynamics property of each identified protein–ligand complex and was compared with the dynamic behavior of the ligand-free protein (LFP) co-crystalline inhibitor bound protein. The average values of every parameter calculated from molecular dynamics (MD) trajectories has been depicted in Table 3.

**Table 3:** Average values of Root Mean Square Deviation (RMSD), Root Mean Square Fluctuation (RMSF), Radius of Gyration (Rg), Solvent Accessible Surface Area (SASA), MM/PBSA based binding free energy calculated from 100 ns molecular dynamics trajectories.

Sl no	Compound ID	Average RMSD (Å)	Average RMSF (Å)	Average Rg (Å)	Average SASA (Å^2^)	Binding energy (kj/mol)
1	Apo protein	2.38	1.34	22.51	1508.6	–
2	Baicalein*	–	–	–	–	–
3	M_78	2.42	1.05	22.43	1483.26	− 193.55 ± 4.8
4	M_82	2.20	1.06	22.42	1460.12	− 180.10 ± 2.6
5	M_83	2.25	1.14	22.16	1493.40	− 174.73 ± 4.4
6	M_88	3.18	1.46	22.75	1520.00	− 177.73 ± 6.2
7	M_111	2.60	1.38	22.56	1518.49	− 147.71 ± 89
8	M_112	1.50	1.07	22.45	1517.50	− 146.60 ± 2.9
9	M_201	2.52	3.13	22.40	1456.00	− 153.50 ± 7.69
10	M_366	1.98	1.18	22.50	1486.14	− 190.46 ± 0.18

*Data available in Supplementary^81^.

**Figure 2:**
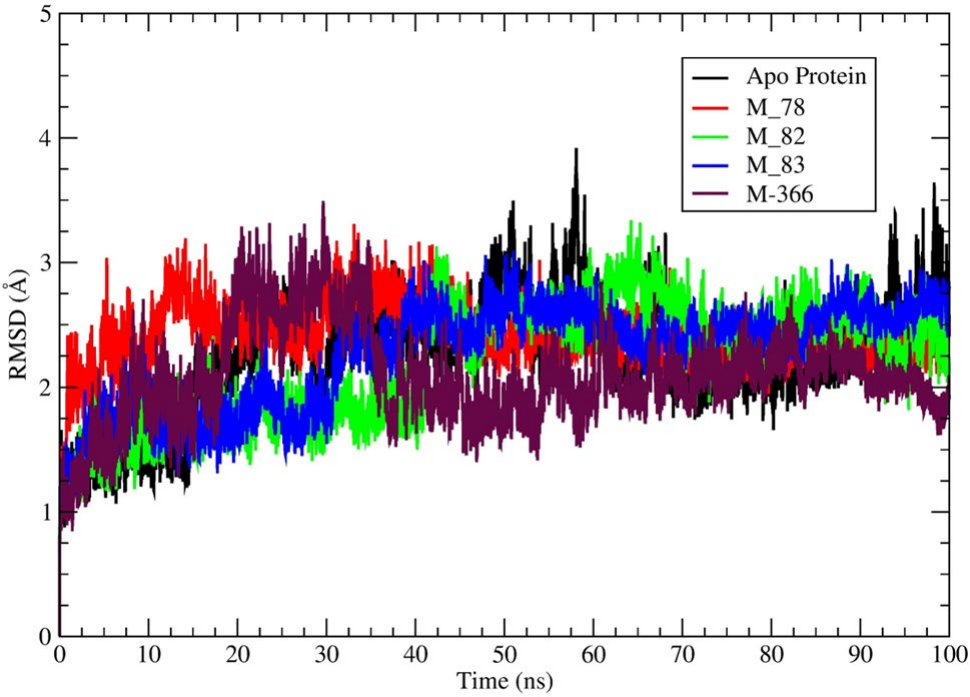
A visual representation of M^pro^ backbone RMSD (Å) of M^pro^‒ligand (M_78, M_82, M_83, M_366) complexes and apo-protein obtained from 100 ns MD simulation trajectories. Different ligands represented by different colours.

The acceptable average RMSD value for globular protein is ≤ 3.0Å^80^. Nonetheless, lower RMSD values are ideally acceptable. It was observed that the protein backbones average RMSD of the selected M^pro^‒ligand (M_78, M_82, M_83, M_111, M_112, M_201, M_366) were less than 3.0 Å (Table 3). The average RMSD value exhibited by M_82, M_83, M_112, M_201, and M_366‒protein systems was lower than the RMSD value of apo-protein. The average RMSD of M_366‒protein system was found to be ~ 1.98 Å, which was even lower than the standard inhibitor baicalein (~ 2.15 Å)^81^. The RMSD profile of the protein-M_88 system was > 3.0 Å. To compare the RMSD of the protein backbone profile of protein–ligand (M_78, M_82, M_83, M_366) system with apo-protein, the RMSD was plotted against time from the 100 ns molecular dynamics trajectories as shown in Fig. 2. Similarly, the RMSD profiles of the protein–ligand (M_88, M_111, M_112, M_201) system has been depicted in Fig. S5.

**Figure 3:**
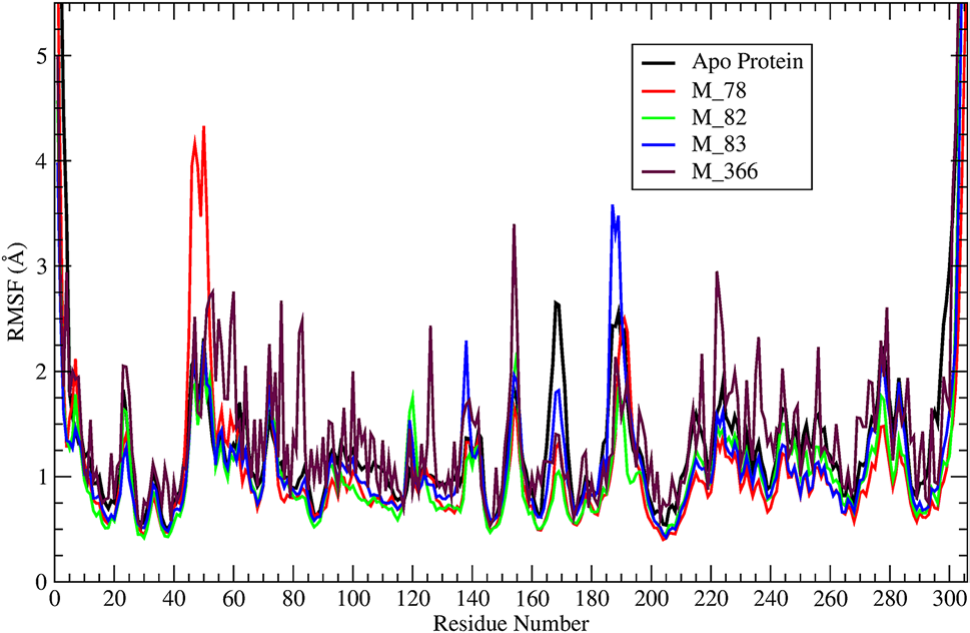
A visual representation of M^pro^ backbone RMSF vs residue number of M^pro^ –ligand (M_78, M_82, M_83, M_366) systems and apo-protein during 100 ns simulation.

To analyze the fluctuation of the individual amino acid residues, the RMSF parameter was calculated for each protein–ligand complex system from the 100 ns molecular dynamics trajectories. Lower the RMSF value generalizes that after binding with the ligand, the fluctuation of the amino acid residues under consideration is reduced. These facts infer stable protein ligands binding, i.e., after binding with ligand, the fluctuation of residues is minimized. The amino acid residues THR-24 to GLN-192 lies in the binding site region of this protein. Amongst the residues, the HIS-41 and CYS-145 are the important residues that regulate the functionality of this protein^78^. The average RMSF value of each protein–ligand system has been mentioned in Table 3. The plots of protein–ligand (M_78, M_82, M_83, M_366) system amino acid residues and RMSF has been shown in Fig. 3. Similarly, the plots of protein–ligand (M_88, M_111, M_112, M_201) system residue vs RMSF have been depicted in Fig. S6.

It can be ascertained from Table 3 that each system other than M_88 and M_201 bound system, exhibited lower RMSF values in comparison to the apro-protein RMSF. In contrast, M_111 system depicted a slightly higher RMSF (~ 1.38 Å) value. Each residue of M_88 and M_201 system showed significantly higher RMSF profiles (Fig. S6). Visual inspection of the trajectory concluded unacceptable changes taking place in the M_201 bound system during the 100 ns simulation time. The radius of gyration parameter further confirmed that event.

**Figure 4:**
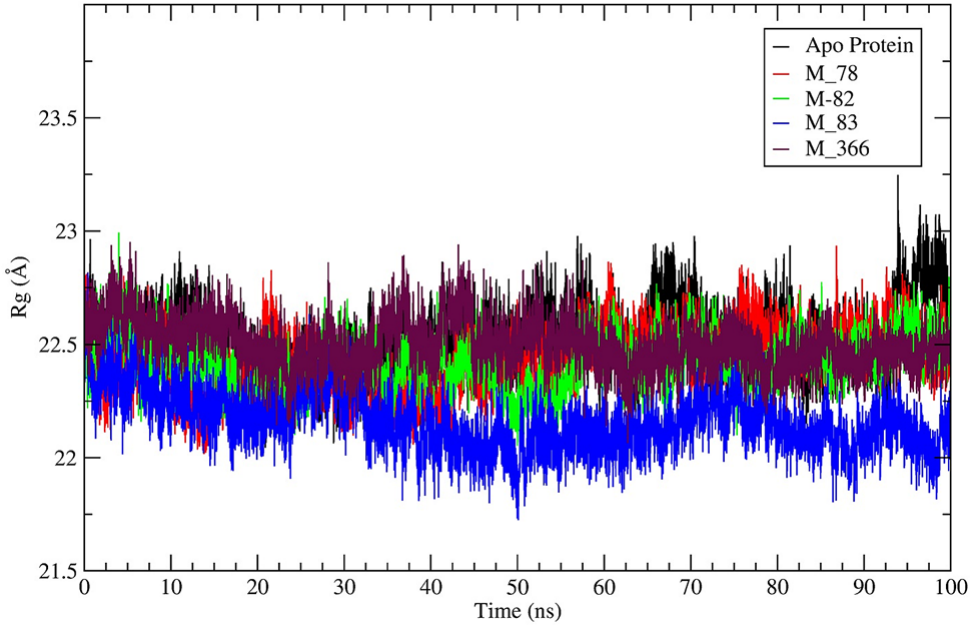
A visual representation of radius of gyration (Rg) vs time of M^pro^‒ligand (M_78, M_82, M_83, M_366) systems and apo-protein during 100 ns MD simulation.

The parameter radius of gyration (Rg) furnishes information about the compactness of the protein. The higher value of Rg indicates that the protein changes its conformation of distortion during the simulation. The average Rg value of the protein–ligand (M_78, M_82, M_83, M_112, M_201, M_366) system was lower than the Rg value of apo-protein (Table 3). The average Rg value of the protein–ligand (M_78, M_82, M_83, M_201) system was lower than the Rg value of the standard inhibitor (22.44 Å). The Rg profile of the hits M_78, M_82, M_83, and M_366 have been depicted in Fig. 4 and Rg profile of M_88, M_111, M_112, M_201 has been shown in Fig. S7.

**Figure 5:**
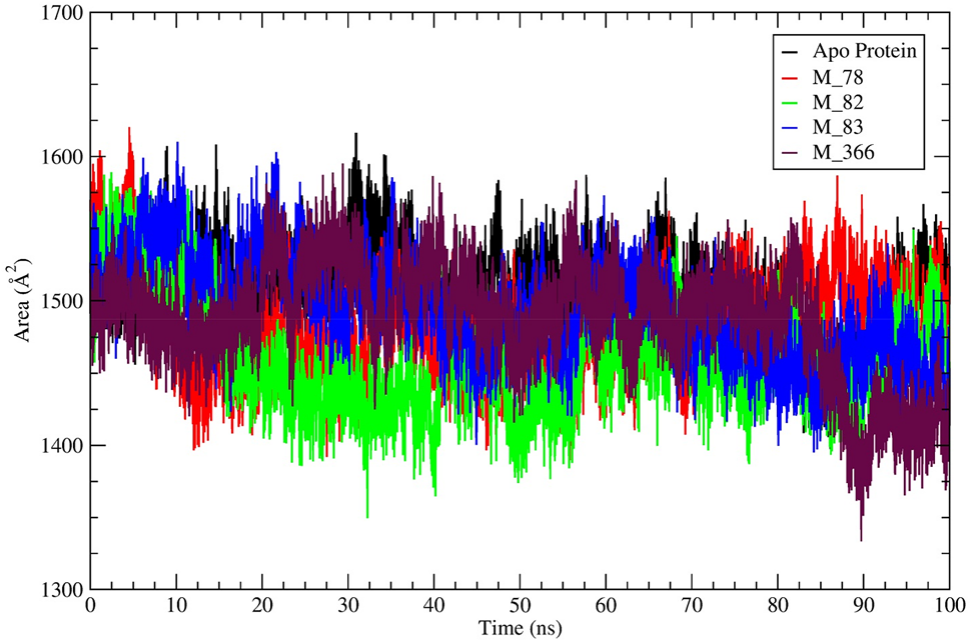
A visual representation of solvent accessible surface area (SASA) of M^pro^‒ligand (M_78, M_82, M_83, M_366) systems and apo-protein during 100 ns simulation time.

The protein–ligand system's solvent-accessible surface area parameter (SASA) was calculated for each protein–ligand system and plotted against the time in nanosecond. Considering that ligand binding is a solvent replacement process, the lower values of the SASA parameter indicate that the binding pocket is less solvent-exposed, and the ligand retains inside the binding pocket during the simulation. The average SASA value of the protein–ligand (M_78, M_82, M_83, M_201, M_366) system was lower than the SASA value of apo-protein (Table 3). The average SASA value of the protein–ligand (M_82, M_201) system was lower than the SASA value of the standard inhibitor (1472Å2). The changes in SASA of M^pro^‒ligand (M_78, M_82, M_83, M_366) systems and apo-protein during 100 ns simulation time have been shown in Fig. 5. Similarly, the changes of SASA value of M_88, M_111, M_112, M_201 protein system have been shown in Fig. S8. Each protein–ligand system, other than a protein–ligand system of M_111, and M_112, showed a lower average SASA value in comparison to the apo-proteins average SASA value (Table 3). Nevertheless, the M_201 system exhibited a SASA value of ~ 1456.0Å^2^ (lower than the apo-protein SASA value) which indicated that the ligand M_201 resided deeply into the binding pocket. However, this binding caused significant changes in the protein structure.

It was observed from Fig. S9 that the center of mass (CoM) distance for all the ligands resided within ~ 5.0 Å distance from the main protease binding site. The hits M_78 and M_82 consistently depicted ~ 2.0 Å distance from the protein binding pocket throughout the simulation. Ligand M_83 after ~ 85^th^ ns showed slightly higher displacement (~ 1.0 Å). After ~ 95 ns, the protein binding pocket M_83 distance reduced to ~ 2.2 Å. The M_366 after ~ 36^th^ ns showed a higher distance (~ 4.1 Å) from the protein binding pocket. However, after ~ 90 ns the distance reduced and stabilized at ~ 2.8 Å.

### Binding free energy (∆G_bind_)

The MM/PBSA based binding free energy (∆G_bind_) was calculated from the total 100 ns of the molecular dynamics trajectories. The ∆G_bind_ of the standard inhibitor was − 180.50 kJ/mol, and the ∆G_bind_ of M_78, M_201, and M_366 were − 193.55, − 193.50 − 190.10 kj/mol, respectively, greater than the ∆G_bind_ of the standard inhibitor (Table 3). The ∆G_bind_ of M_82 was − 180.10 kj/mol, which was nearly the same as the ∆G_bind_ value of the standard inhibitor. The ∆G_bind_ value of M_112 and M_111 was low in comparison to the ∆G_bind_ value of the standard inhibitor. Finally, the RMSD value of the hit M_88 was greater than the acceptable range and therefore was not considered as a promising hit. Due to the low ∆G_bind_ value of the hits, M_111 and M_112 which were − 147.71 and − 146.60 kj/mol and greater SASA values in comparison to the apo-protein, the hits M_111 and M_112 were excluded from the list of promising hits. The per-frame binding energy over the simulated time of M_78, M_82, M_83, and M_366 are depicted in Fig. S10. The RMSD value of hit M_201 was comparatively high (2.52 Å), and its ∆G_bind_was also comparatively low and therefore excluded from the list of promising hits. From the analysis of various parameters like RMSD, RMSF, Rg, SASA, and MM/PBSA calculated from the MD trajectories, it can be stated that the ligand M_78, M_82, M_83, and M_366 might have the potential to form a stable complex with SARS-CoV-2M^pro^.

**Figure 6:**
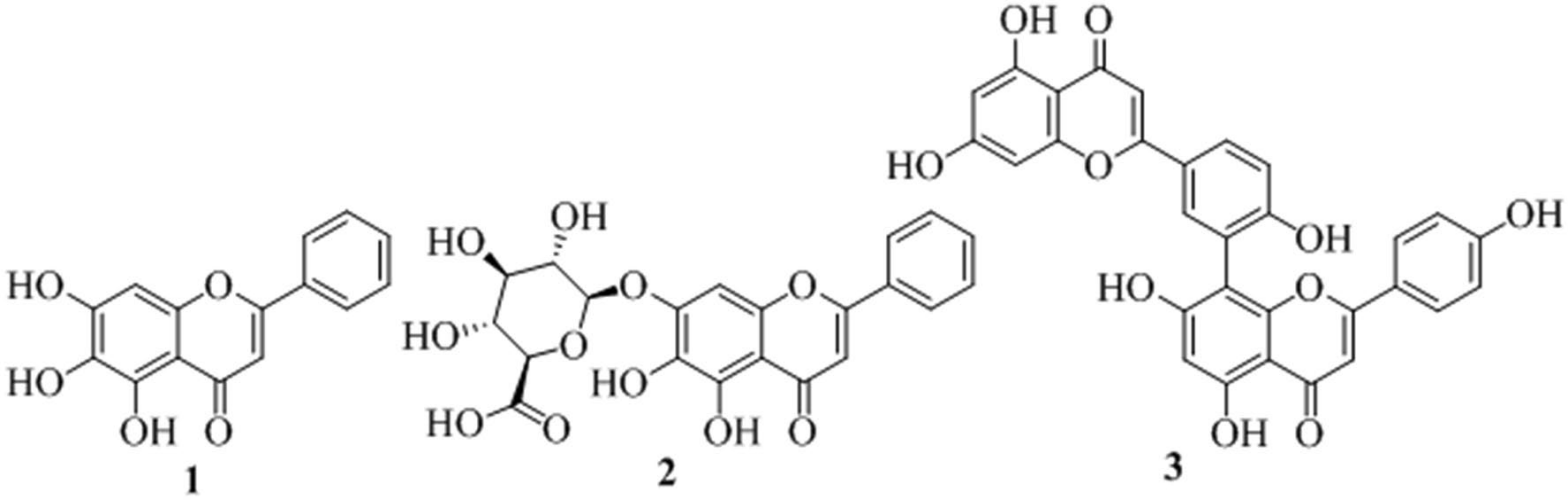
Structures of three known SARS-CoV-2 flavone inhibitors **1** (baicalein), 2 (baicalin) and **3** (biflavonoids).

There have been a large report of antiviral activities of mushroom compounds (Table S3) and therefore identification of SARS-CoV-2 M^pro^ inhibitors from reported mushroom compounds is of great interest. All the proposed compounds are novel, structurally diverse, and contain phenolic functional groups. Polyphenolic compounds perform a series of defensive activities in the human body. One of the important classes of phenolic compounds are flavonoids, which showed blocking potential against different viral proteins like M^pro^, PL^pro^, Spike against SARS-CoV and MARS-CoV^82^. Two flavones found in different plants, baicalein (**1**) and baicalin (**2**) (Fig. 6), exhibited in vitro inhibition against SARS-CoV-2 M^pro^ and IC_50_ values, of which were 6.41 and 0.94 μM, respectively^83^. Another biflavonoids (**3**) found in *Torreyanucifera* showed inhibitory activity against SARS-CoVM^pro^ and the IC_50_ value of which was 8.3 μM^84^.

The ADV docking scores of the proposed hits were similar to the ADV docking score of the known inhibitors **1**, **2**, and **3** and the docking scores were ‒7.7, ‒8.6, and ‒8.7 kcal/mol, respectively (Table S1). The best docking poses of the proposed hits M_78, M_82, M_83, and M_366 in the active site superimposed on the two SARS-CoV-2 known inhibitors **1** and **2** have been shown in Fig. S3. It was observed that the pyran ring, chromen-4-one-oxygen atom, pyran-1-oxygen atom of inhibitors **1** matched with the middle ring, an acetylated-phenolic-oxygen atom of the middle ring, and phenolic-OH of the middle ring, respectively, of hit M_78. Two rings of one of the benzofuran moiety; 5,6-dihydroxy group of one of the benzofuran moiety, furan oxygen of benzofuran moiety of hit M_82 matched with two rings of inhibitor **1**; a 6,7-dihydroxy group of **1**, and pyran-1oxygen of **1**, respectively. The matching of the pharmacophore of hit M_83 was very close to M_82. The pyran ring, 6-hydroxyl group, and pyran-1-oxygen of compound **1** matched with one of the aromatic rings of dibenzofuran, 1-hydroxy group of dibenzofuran, and furan oxygen, respectively with M_366. The matching of the pharmacophores of the proposed hits in the most stable binding pose with standard inhibitor baicalein was again a piece of evidence in the favour of the potential of the selected hits, against SARS-CoV-2 M^pro^.

## Conclusion

Based on the detailed in-silico studies, it can be concluded that compounds M_78, M_82, M_83, and M_366 showed docking scores greater than co-ligand and comparable docking scores with the two known SARS-CoV-2 inhibitors. The binding affinity of the hits M_78, M_82, M_83, M_366 against M^pro^ protein of SARS-CoV-2 was also supported by all the MD parameters such as RMSD, RMSF, Rg, SASA, MM-PBSA binding energy (∆G_bind_). Moreover, all the proposed hits obeyed the Lipinski rule of five and matches pharmacophores with known inhibitors in the active site. The promising hits Kynapcin-12 (M_78), Kynapcin-28 (M_82), Kynapcin-24 (M_83) are available in edible mushroom *Polyozellus multiplex* and another promising hit Neonambiterphenyls-A (M_366) is available in the poisonous mushroom *Neonothopanus nimbi*. The mushroom *P. multiplex* contains three potential hits which might be used as a remedy against COVID-19 after the appropriate biological screening. These novel phenolic scaffolds may be further developed as more potential SARS-CoV-2 inhibitors.

## Supplementary Information

Supplementary Information 1.

Supplementary Information 2.
