# Supplementary material for: Identification of potential edible mushroom as SARS-CoV-2 main protease inhibitor using rational drug designing approach
Source: Sci Rep. 2022 Jan 27;12:1503. doi: 10.1038/s41598-022-05349-x (PMC8795408; doi:10.1038/s41598-022-05349-x)
Supplement: Supplementary file 1 — Supplementary Information 1. [file 41598_2022_5349_MOESM1_ESM.docx]

**Supplementary Information of the Article**

**Identification of potential edible mushroom as SARS-CoV-2 main protease inhibitor using rational drug design approach**

Debanjan Sen^a^*,* Bimal Debnath^b^, Pradip Debnath^c^, Sudhan Debnath^d*^, Magdi E.A. Zaki^e*^, Vijay H. Masand^f^

*^a^BCDA College of Pharmacy & Technology, Jessore Road South, Hridaypur, Kolkata, West Bengal, 700127, India*

*^b^Department of Forestry and Biodiversity, Tripura University, Suryamaninagar, Tripura, 799022, India*

*c Department of Chemistry, Majaraja Bir Bikram College, Agartala, Tripura, 799004, India*

*^d^Department of Chemistry, Netaji Subhas Mahavidalaya, Udaipur, Tripura, 799114, India*

*^e^Department of Chemistry, Faculty of Science, Imam Mohammad Ibn Saud Islamic University, Riyadh 13318, Saudi Arabia.*

*^f^Department of Chemistry, Vidya Bharati Mahavidyalaya, Amravati, Maharashtra, India-444 602*

*Authors for correspondence mail: [bcsdebnath@gmail.com](mailto:bcsdebnath@gmail.com), [*Mezaki@imamu.edu.sa*](mailto:Mezaki@imamu.edu.sa)

| **Contents** |
| --- |
| **Table S1**. Compounds of mushrooms and their activity |
| **Table S2**. Structure of known SARS-CoV-2 inhibitors (**1**, **2, 3**), docking score predicted by AutoDock Vina and AutoDock 4.2 |
| **Figure S1**. 2D ligand binding interaction analysis of selected hits (M_78, M_82, M_83, M_88, M_111, M_112, M_201, M_366, M_421, M_505 and coligand) with SARS-CoV-2 main protease (PDB ID: 6LU7) |
| **Figure S2**. Binding pose in 3D space of selected hits (yellow) in the receptor binding domain of SARS-CoV-2 M^pro^ |
| **Figure S3**. Comparison of binding pose of selected hits (M_78, M_82, M_83, M_88, M_111, M_112, M_336) with known inhibitors **1**, **2** in the active site of M^pro^ |
| **Figure S4**. M^pro^ backbone RMSD of M^pro^‒ligand (M_88, M_111, M_112, M_201) complex and apo-protein obtained from 100 ns MD simulation trajectories |
| **Figure S5**. M^pro^ backbone RMSF vs residue number of M^pro^ –ligand (M_88, M_111, M_112, M_201) system and apo-protein during 100 ns simulation  **Figure S6**. Radius of gyration vs time during 100 ns MD simulation of M^pro^‒ligand (M_88, M_111, M_112, M_201) systems and apo-protein |
| **Figure S7**. Solvent accessible surface area of M^pro^‒ligand (M_88, M_111, M_112, M_201) systems and apo-protein during 100 ns simulation time |
|  |

| **Table S1**. Reported antiviral activities of mushroom compounds | | |
| --- | --- | --- |
| Name of compounds | Sources | Activity against Reference |
| 1. Triterpines applanoxidic acid G, lucidadiol, and Ganodermadiol | Ganoderma species | Against influenza virus A and HSV 1 [1] |
| 2. Polysaccharide krestin (PSK) | *Trametesversicolor* (Turkey tail Mushroom) and *Lentinusedodes* mycelium | Extract inhibits cell to cell infection by HIV-1 and HIV-2  [2] |
| 3. Agrocybone | Agrocybesalicacola | Showed antiviral activity against respiratory syncytial virus (RSV) [3] |
| 4. Ganoderic acid, a triterpene from | *Ganodermalucidum* | Inhibits the replication of hepatitis B virus [4] |
| 5. Ganoderic acid A–C, ganoderic acid β, ganolucidic acid A, lucidumol B, ganodermanontriol, 3β-5α-dihydroxy-6β-methoxyergosta-7,22-diene, ganodermanondiol found in ganomycin I, colossolactones (A, E, G, V, VII, VIII), ganomycin-B from *G. colosum*; ganoderiol A, ganoderiol F, 20-hydroxylucidenic acid N, ganoderic acid GS-2, 20(21)-dehydrolucidenic acid N, lucidumol A | *Ganodermalucidum*; *G. sinnense* | Potential HIV-1protease inhibition activity at µM range [5, 6, 7, 8] |
| 6. Lactinsconcanavalin A | *Volvariellavolvacea* | activate T lymphocytes [9] |
| 7. Ricin-B-like lectin (CNL) | *Clitocybenebularis* | Stimulating dendritic cells (DCs) and cytokines. [9] |
| 8. Terpenoids, exobiopolymers | *Ganodermaapplanatum* | Activate Natural Killer (NK) cells, [9] |
| 9. Ganolucidoid A and B | Ganodermalucidum | Anti-inflamatory activity |
| 10. lanostane | (Hypholomafasciculare) | exhibit anti-inflamatory activity [9] |

| **Table S2**. Structure of known SARS-CoV-2 inhibitors, and their docking score predicted by AutoDock Vina and AutoDock 4.2 | | | | |
| --- | --- | --- | --- | --- |
| Compound | Structure | *AVDS  kcal/mol | ^#^ADS  kcal/mol | Ki |
| **1** |  | -7.7 | -7.29 | 4.49 μM |
| **2** |  | -8.6 | -7.63 | 2.53 μM |
| **3** |  | -8.7 | -10.1 | 35.13 nM |
| ***AVDS**= AutoDock Vina Docking Score, **^#^ADS**= AutoDock4.2 Docking Score | | | | |


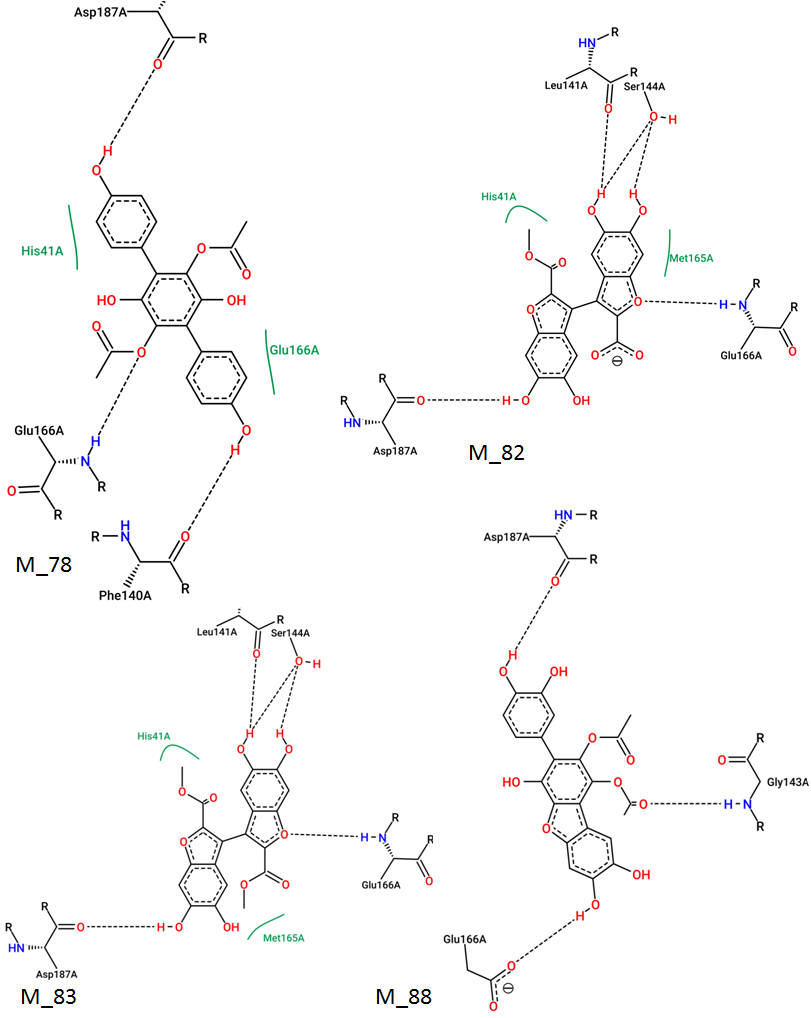


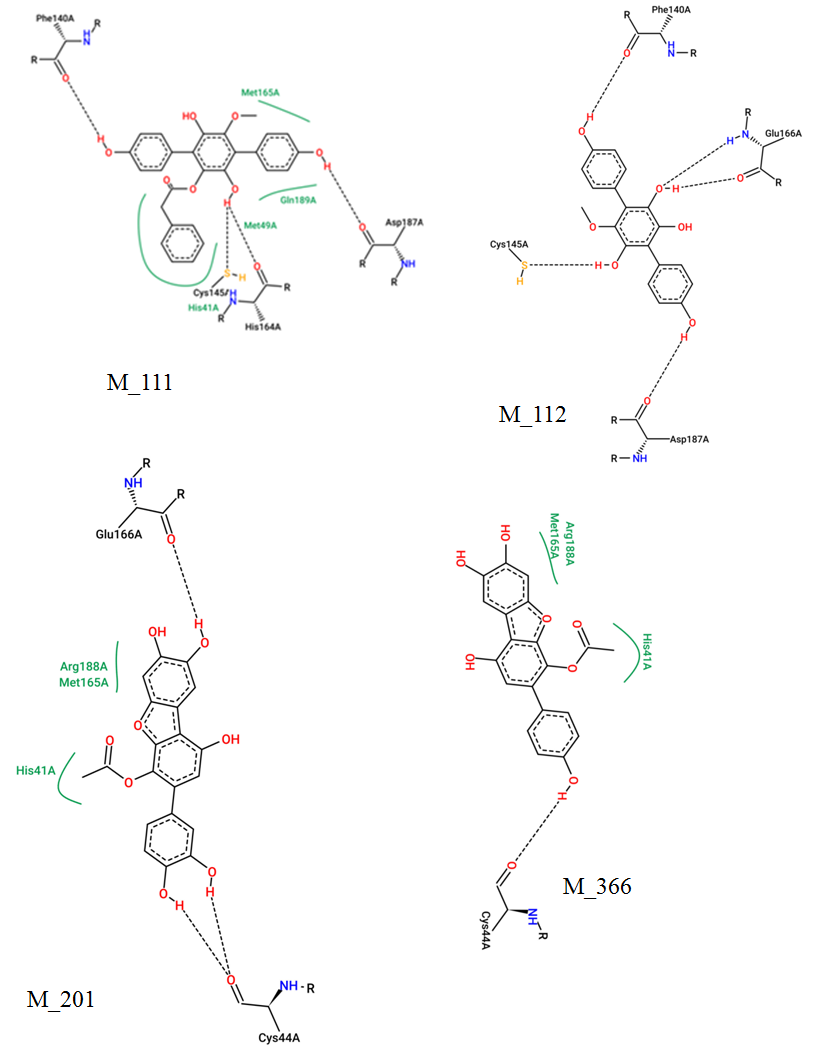


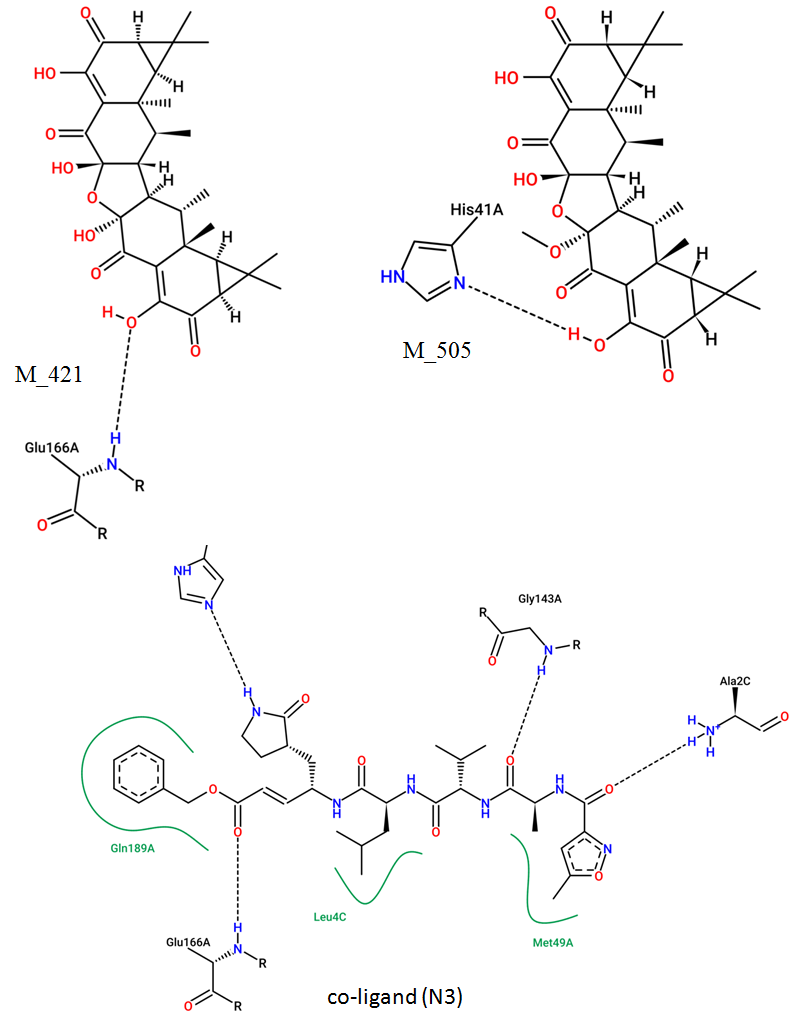


**Figure S1**. 2D ligand binding interaction analysis of selected hits (M_78, M_82, M_83, M_88, M_111, M_112, M_201, M_366, M_421, M_505 and coligand) with SARS-CoV-2 main protease (PDB ID: 6LU7)


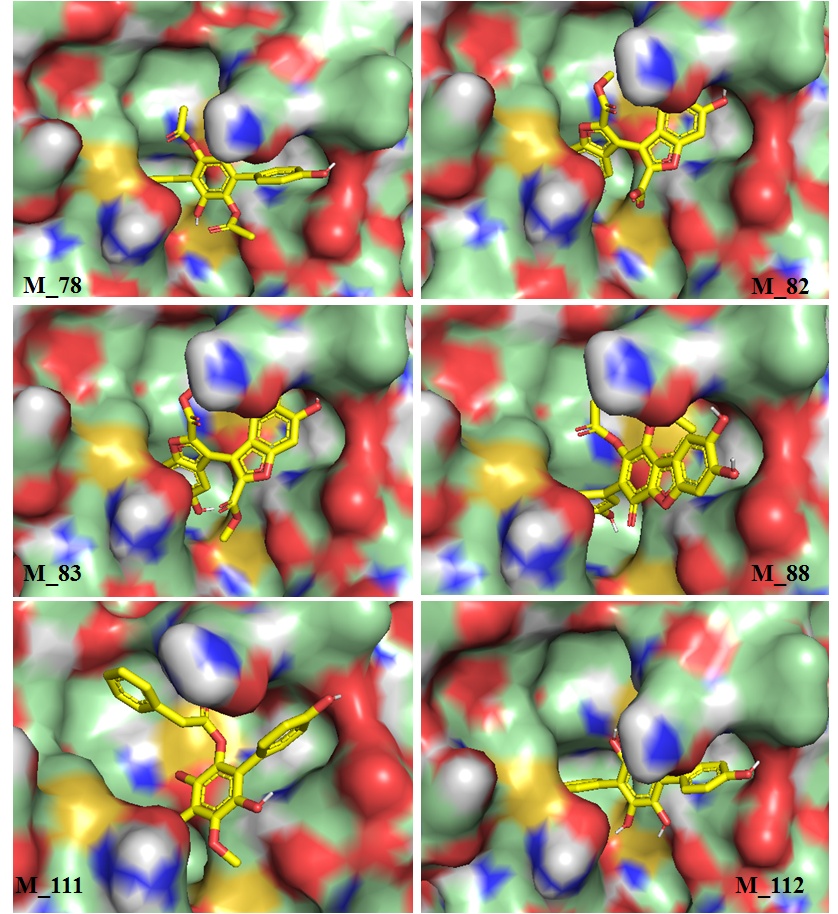


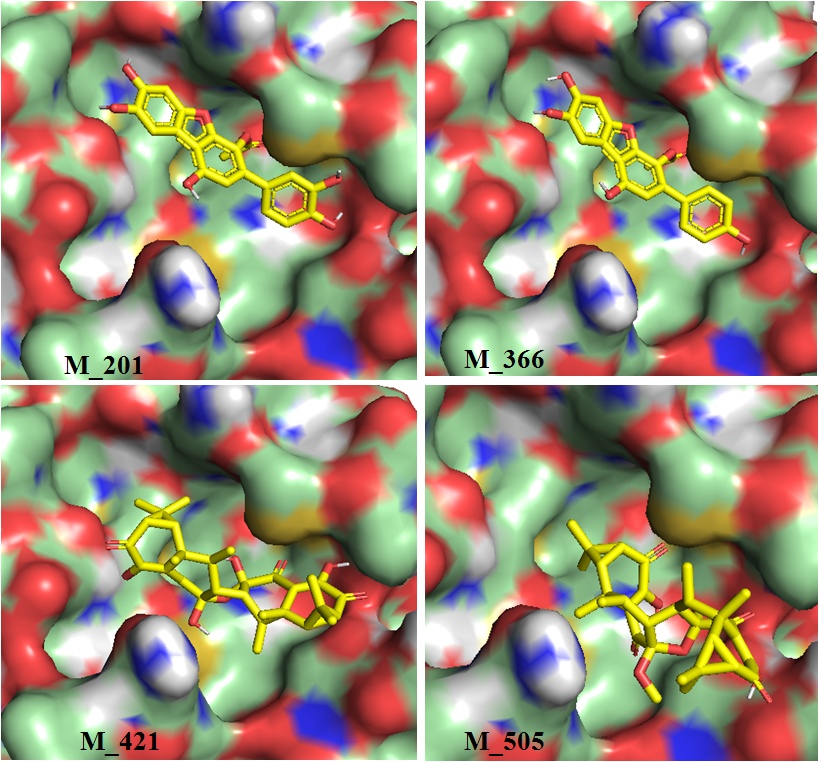


**Figure S2**. Binding pose in 3D space of selected hits (yellow) in the receptor binding domain of SARS-CoV-2 M^pro^


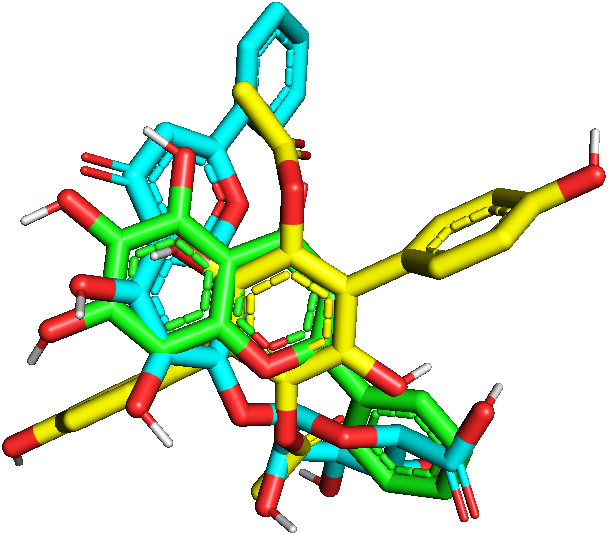


M_78 (yellow)-**1**-(green)-**2**-(sky) in the active site


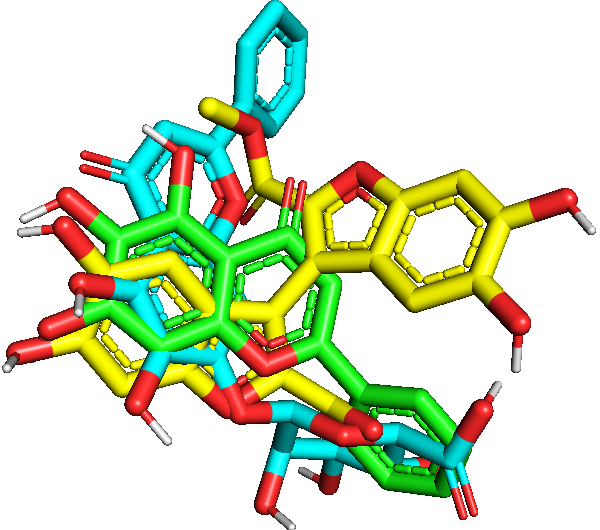


M_82 (yellow)-**1**-(green)-**2**-(sky) in the active site


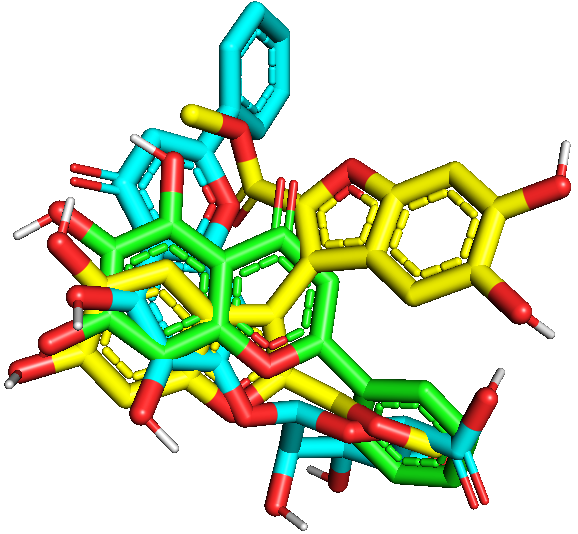


M_83 (yellow)-**1**-(green)-**2**-(sky) in the active site


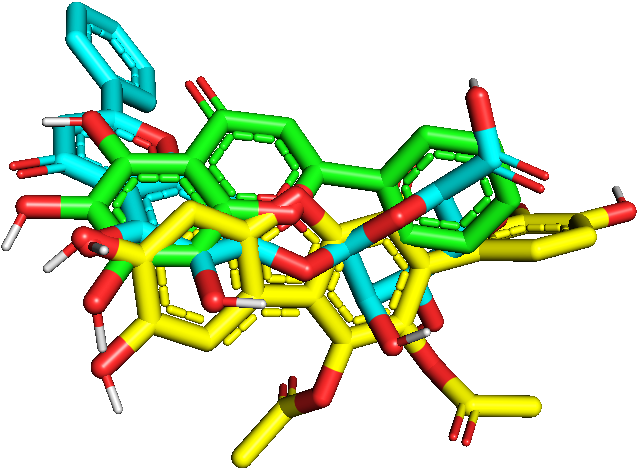


M_88 (yellow)-**1**-(green)-**2**-(sky) in the active site


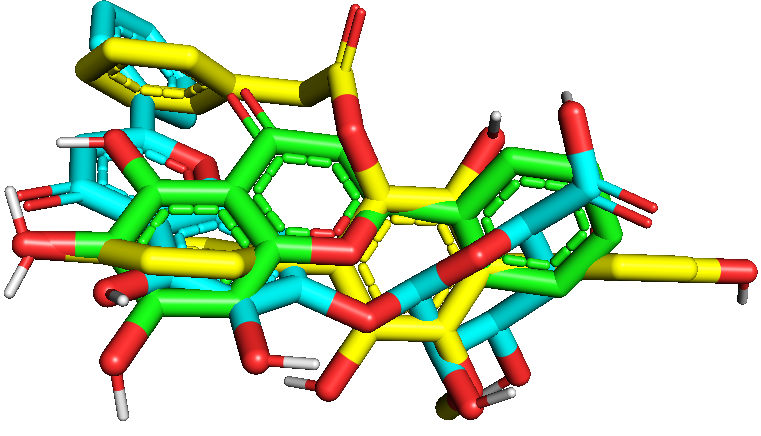


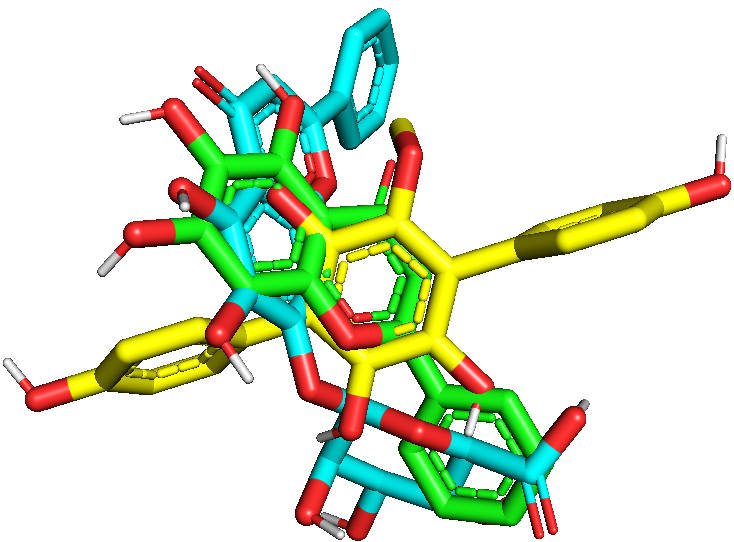
M_111 (yellow)-**1**-(green)-**2**-(sky) in the active site

M_112 (yellow)-**1**-(green)-**2**-(sky) in the active site


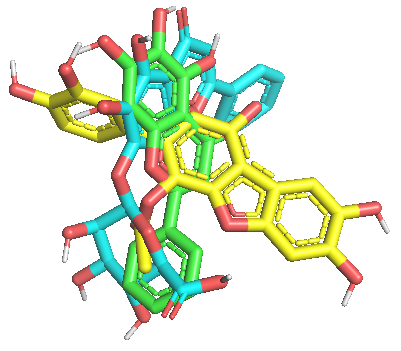


M_201 (yellow)-**1**-(green)-**2**-(sky) in the active site


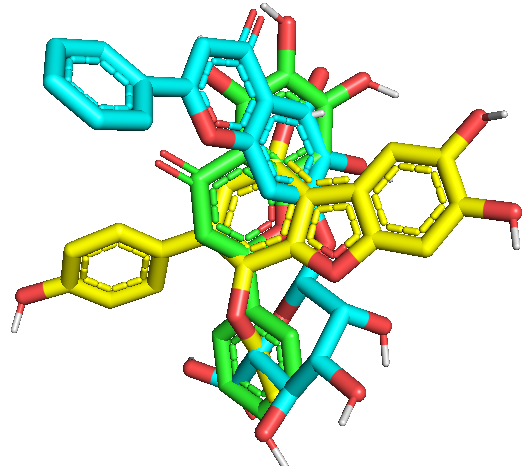


M_336 (yellow)-**1**-(green)-**2**-(sky) in the active site

**Figure S3**. Comparison of binding pose of selected hits (M_78, M_82, M_83, M_88, M_111, M_112, M_336) with known inhibitors **1**, **2** in the active site


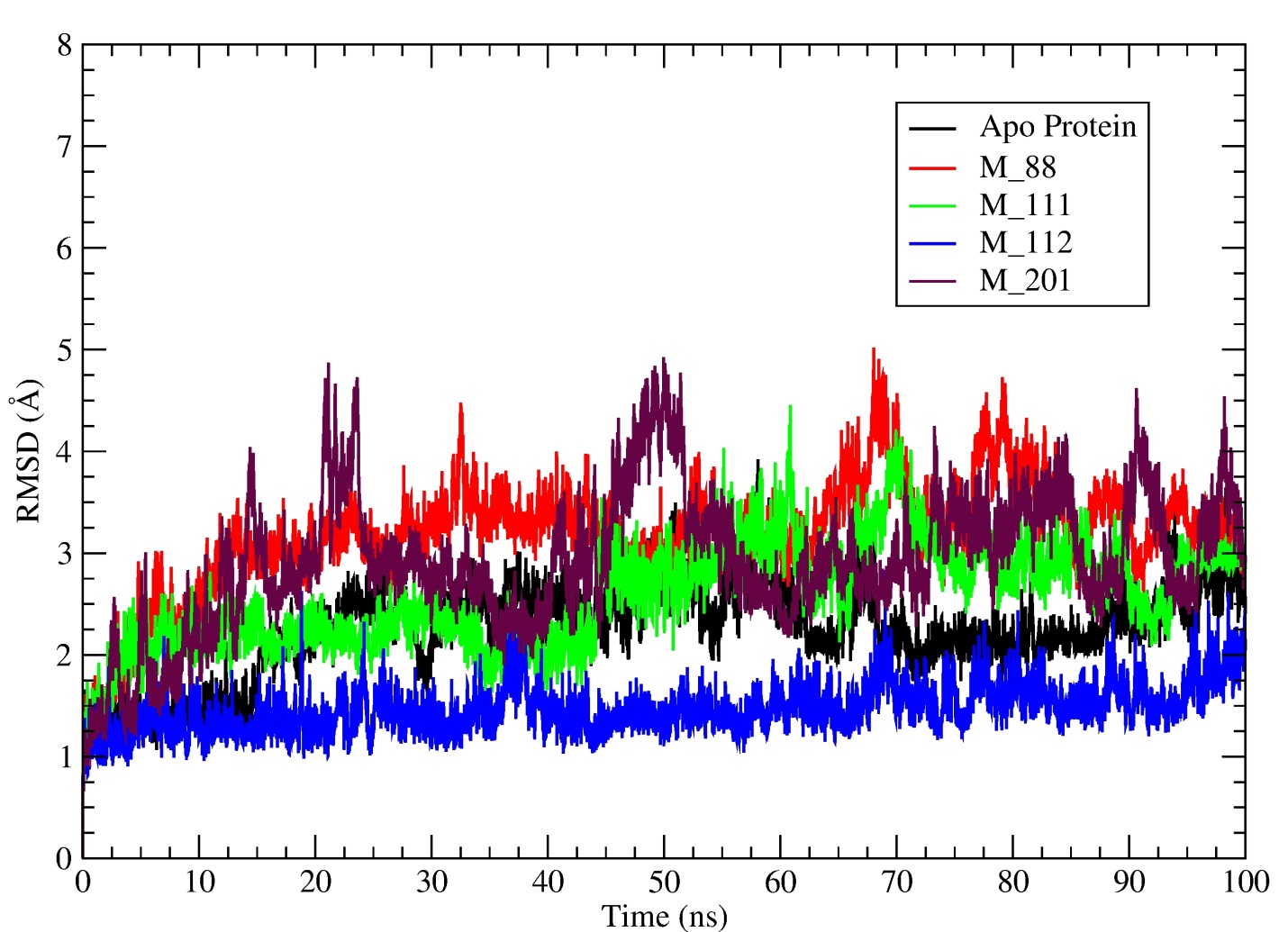


**Figure S4**. M^pro^ backbone RMSD of M^pro^‒ligand (M_88, M_111, M_112, M_201) complex and apo-protein obtained from 100 ns MD simulation trajectories


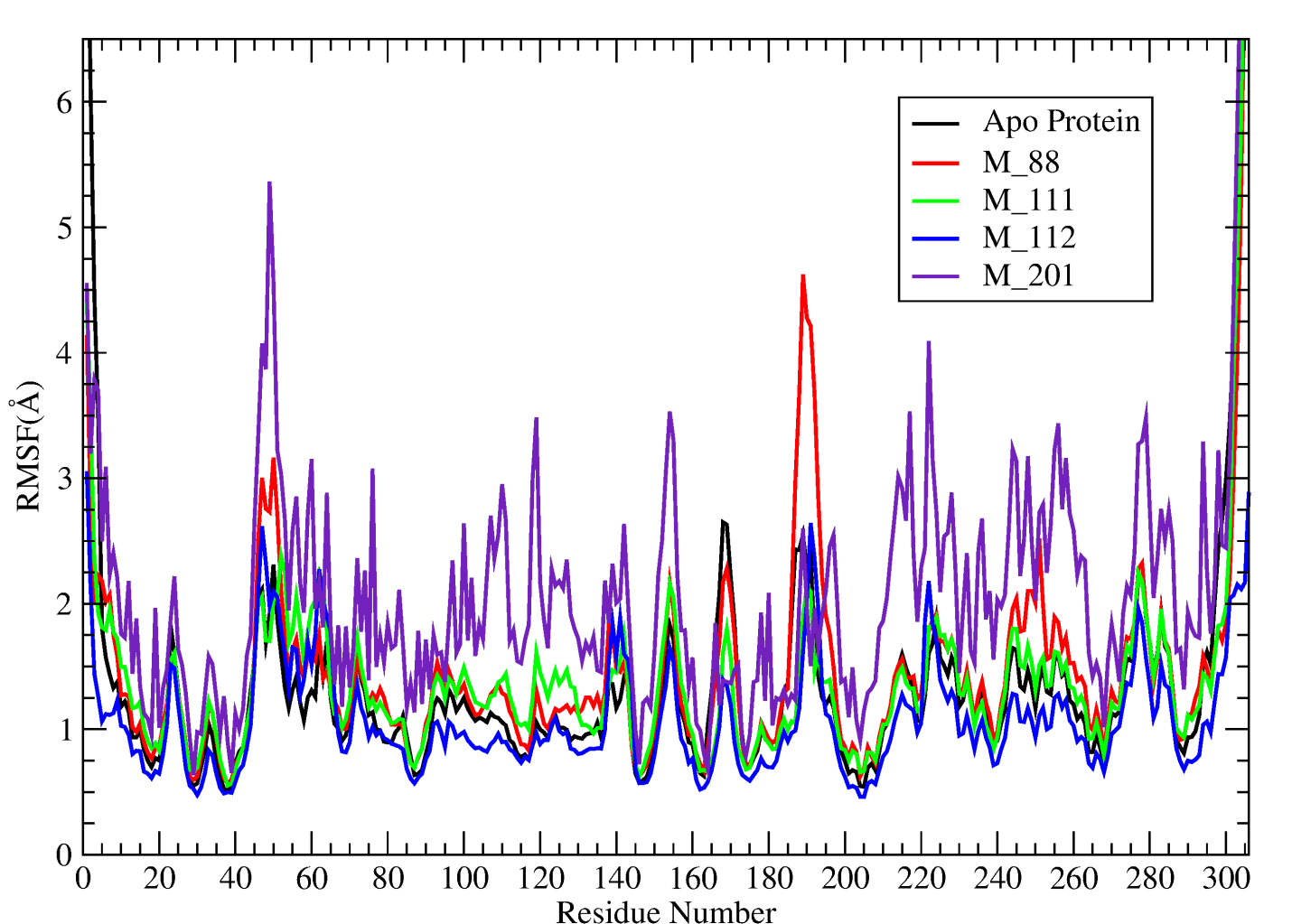


**Figure S5**. M^pro^ backbone RMSF vs residue number of M^pro^ –ligand (M_88, M_111, M_112, M_201) system and apo-protein during 100 ns simulation


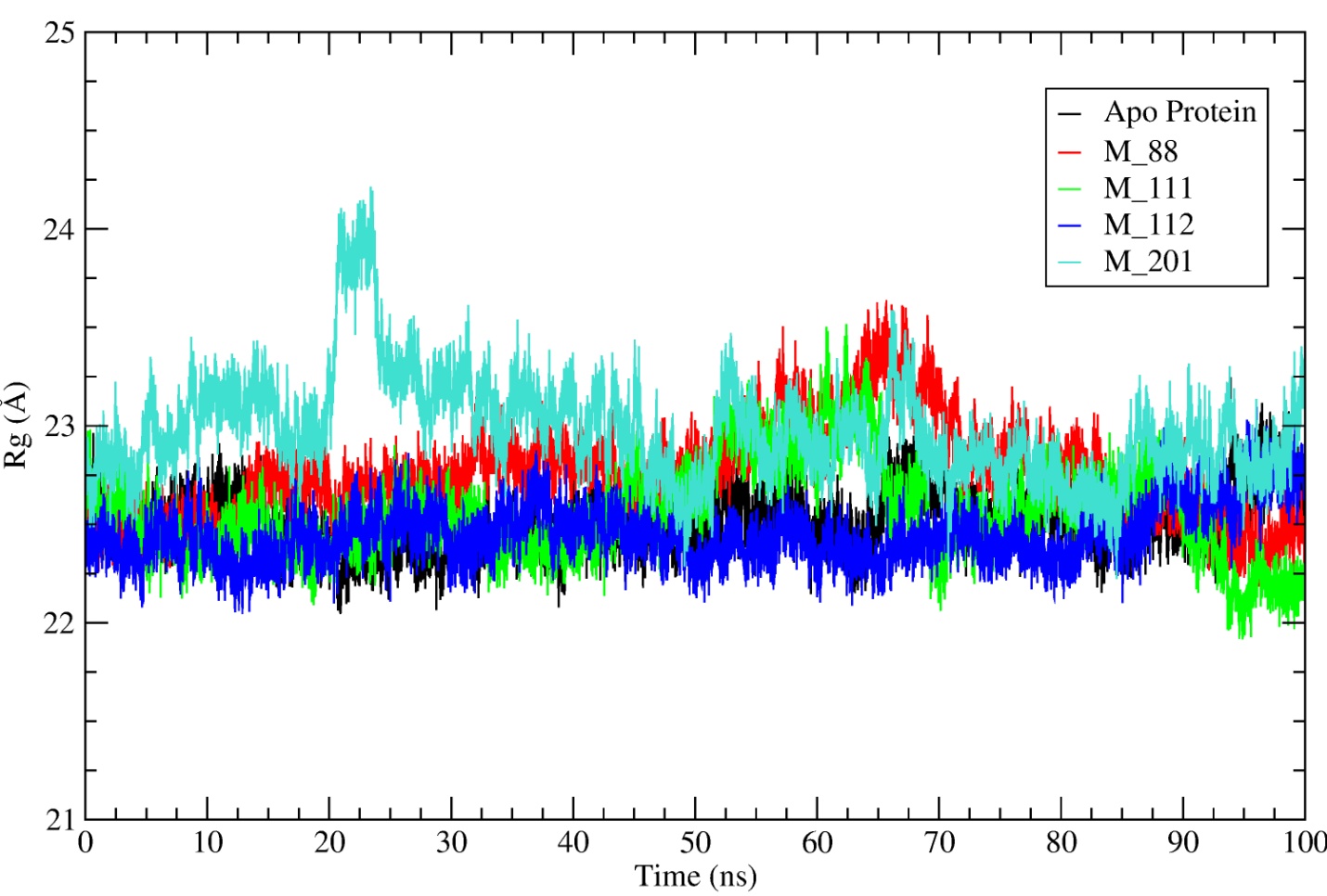


**Figure S6**. Radius of gyration vs time during 100 ns MD simulation of M^pro^‒ligand (M_88, M_111, M_112, M_201) systems and apo-protein


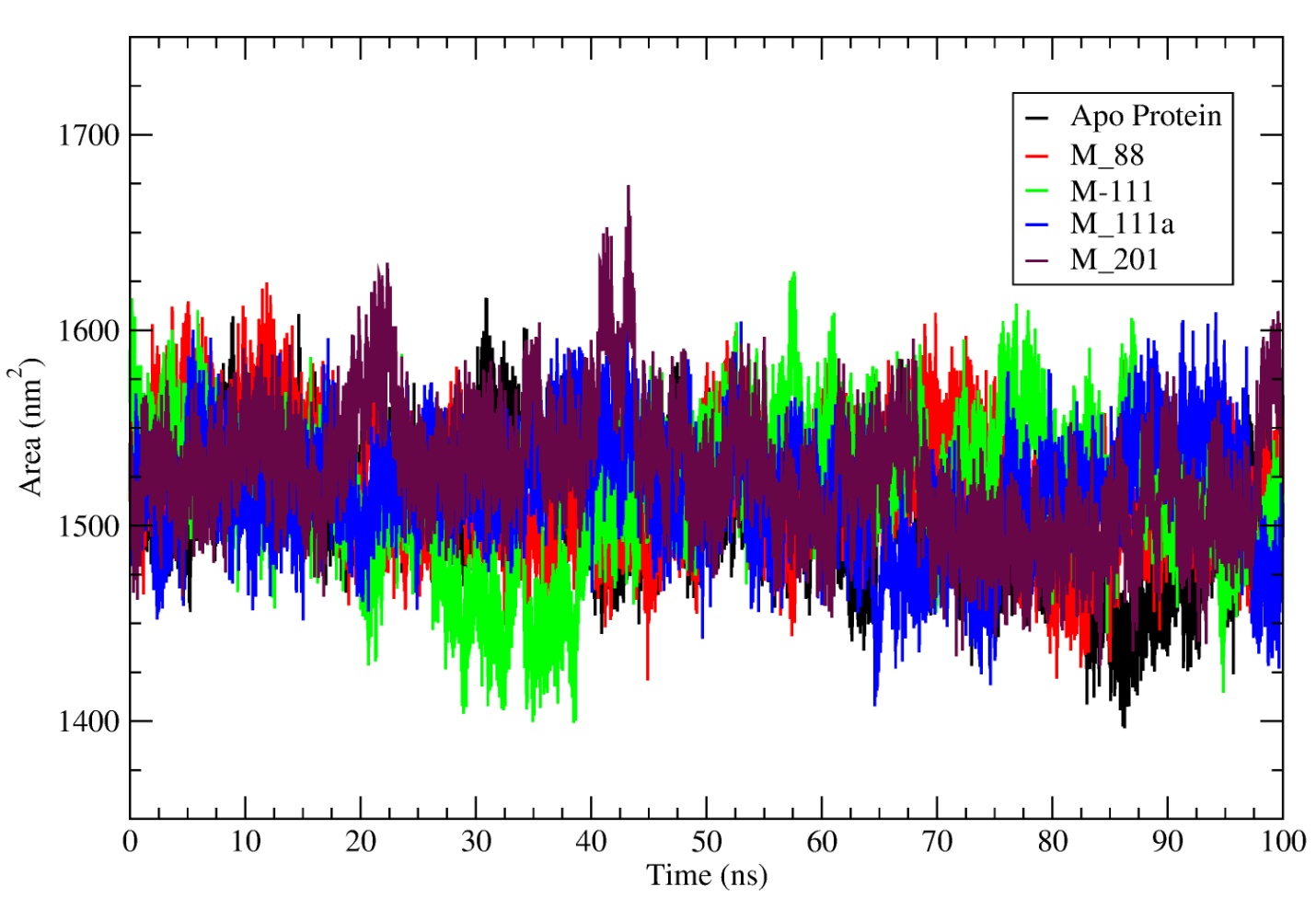


**Figure S7**. Solvent accessible surface area of M^pro^‒ligand (M_88, M_111, M_112, M_201) systems and apo-protein during 100 ns simulation time

**References**

1. Mothana RA, Awadh NA, Jansen R, Wegner U, Mentel R, Lindequist U.2003.Antiviral lanostanoidtriterpenes from the fungus *Ganodermapfeifferi* Bres. Fitoterapia. 74: 177–180

2. Tochikura TS, Nakashima H, Yamamoto N. 1989.Antiviral agents with activity against human retroviruses. *J Acquir Immune DeficSyndr (1988)*. 2(5):441-447

3. Zhu YC, Wang G, Yang XL, Luo DQ, Zhu QC, Peng T, Liu JK. 2010.Agrocybone, a novel bis-sesquiterpene with a spirodienone structure from basidiomyceteAgrocybesalicacola. TetrahedrLett. 51: 3443–5.

4. Li YQ, Wang SF. 2006. Anti-hepatitis B activities of ganoderic acid from *Ganodermalucidum*. *Biotechnollett*. *28(11)*, 837-841. <https://doi.org/10.1007/s10529-006-9007-9>

5. Min BS, Nakamura N, Miyashiro H, Bae KW, Hattori M. 1998. Triterpenes from the spores of *Ganodermalucidum* and their inhibitory activity against HIV-1 protease.*Chem. Pharm. Bull. 46(10)*, 1607–1612. https://doi.org/10.1248/cpb.46.1607

6. El Dine RS, Halawany AME, Ma CM, Hattori M. 2008. Anti-HIV1- protease activity of lanostanetriterpenes from the Vienamese mushroom *Ganodermacolossum*. *J. Nat. Prod*. *71(6)*, 1022–1026. https://doi.org/10.1021/np8001139

7. El Dine RS, El-Halawany A, Ma CM, Hattori,M. 2009.Inhibition of the dimerization and active site of HIV-1 protease by secondary metabolites from the Vietnamese Mushroom *Ganodermacolossum*. *J. Nat. Prod*. *72(11)*, 2019–2023. https://doi.org/10.1021/np900279u

8. Sato N, Zhang Q, Ma CM, Hattori M. 2009. Anti-human immunodeficiency virus-1 protease activity of new lanostane-type triterpenoids from *Ganodermasinense*. *Chem. Pharm. Bull*. *57(10)*, 1076–1080. <https://doi.org/10.1248/cpb.57.1076>

9. Suwannarach N, Kumla J, Sujarit K, Pattananandecha T, Saenjum C, Lumyong S. 2020. Natural Bioactive Compounds from Fungi as Potential Candidates for Protease Inhibitors and Immune modulators to Apply for Coronaviruses. *Molecules*. 25(8):1800. https://doi.org/10.3390/molecules25081800
