# Supplementary material for: Identification of potential edible mushroom as SARS-CoV-2 main protease inhibitor using rational drug designing approach
Source: Sci Rep. 2022 Jan 27;12:1503. doi: 10.1038/s41598-022-05349-x (PMC8795408; doi:10.1038/s41598-022-05349-x)
Supplement: Supplementary file 2 — Supplementary Information 2. [file 41598_2022_5349_MOESM2_ESM.docx]

**Supplementary Information of the Article**

**Identification of potential edible mushroom as SARS-CoV-2 main protease inhibitor using rational drug design approach**

Debanjan Sen^a^*,* Bimal Debnath^b^, Pradip Debnath^c^, Sudhan Debnath^d*^, Magdi E.A. Zaki^e*^, Vijay H. Masand^f^

*^a^BCDA College of Pharmacy & Technology, Jessore Road South, Hridaypur, Kolkata, West Bengal, 700127, India*

*^b^Department of Forestry and Biodiversity, Tripura University, Suryamaninagar, Tripura, 799022, India*

*c Department of Chemistry, Majaraja Bir Bikram College, Agartala, Tripura, 799004, India*

*^d^Department of Chemistry, Netaji Subhas Mahavidalaya, Udaipur, Tripura, 799114, India*

*^e^Department of Chemistry, Faculty of Science, Imam Mohammad Ibn Saud Islamic University, Riyadh 13318, Saudi Arabia.*

*^f^Department of Chemistry, Vidya Bharati Mahavidyalaya, Amravati, Maharashtra, India-444 602*

*Authors for correspondence mail: [bcsdebnath@gmail.com](mailto:bcsdebnath@gmail.com), [*Mezaki@imamu.edu.sa*](mailto:Mezaki@imamu.edu.sa)

| **Contents** |
| --- |
| **Table S1**. Structure of known SARS-CoV-2 inhibitors (**1**, **2, 3**), docking score predicted by AutoDock Vina and AutoDock 4. |
| **Table S2**. Docking score of selected hits with protonated target |
| **Table S3**. Reported antiviral activities of mushroom compounds |
| **Figure S1**. 2D ligand binding interaction analysis of selected hits (M_78, M_82, M_83, M_88, M_111, M_112, M_201, M_366, M_421, M_505 and coligand) with SARS-CoV-2 main protease (PDB ID: 6LU7) |
| **Figure S2**. Binding pose in 3D space of selected hits (yellow) in the receptor-binding domain of SARS-CoV-2 M^pro^ |
| **Figure S3**. Protonated (pH 6-5) target protein (PDB ID: 6LU7) of SARS-CoV-2 M^pro^ |
| **Figure S4**. Comparison of the binding pose of selected hits (M_78, M_82, M_83, M_88, M_111, M_112, M_336) with known inhibitors **1**, **2** in the active site of M^pro^ |
| **Figure S5**. M^pro^ backbone RMSD of M^pro^‒ligand (M_88, M_111, M_112, M_201) complex and apo-protein obtained from 100 ns MD simulation trajectories |
| **Figure S6**. M^pro^ backbone RMSF vs residue number of M^pro^ –ligand (M_88, M_111, M_112, M_201) system and apo-protein during 100 ns simulation  **Figure S7**. The radius of gyration vs time during 100 ns MD simulation of M^pro^‒ligand (M_88, M_111, M_112, M_201) systems and apo-protein |
| **Figure S8**. The solvent-accessible surface area of M^pro^‒ligand (M_88, M_111, M_112, M_201) systems and apo-protein during 100 ns simulation time |
| **Figure S9**. A visual inspection of the protein-ligand (78, 82, 83 and 366) distance i.e. center of mass (CoM) derived from 100 ns simulation time |
|  |
| **Figure S10**. Visual inspection of per frame binding energy over the simulated time |

| **Table S1**. Structure of known SARS-CoV-2 inhibitors **1** (baicalein), **2** (baicalin) and **3** (biflavonoids), and their docking score predicted by AutoDock Vina and AutoDock 4.2 | | | | |
| --- | --- | --- | --- | --- |
| Compound | Structure | *ADVS  kcal/mol | ^#^ADS  kcal/mol | Ki |
| **1** |  | -7.7 | -7.29 | 4.49 μM |
| **2** |  | -8.6 | -7.63 | 2.53 μM |
| **3** |  | -8.7 | -10.1 | 35.13 nM |
| ***ADVS**= AutoDock Vina Docking Score, **^#^ADS**= AutoDock 4.2 Docking Score | | | | |

| **Table S2.** Docking score of selected hits with protonated target (exhaustiveness used during docking = 50) | | | | | |
| --- | --- | --- | --- | --- | --- |
| Sl No | Compound Number | AVD score  kcal/mol | SL No | Compound Number | AVD score  kcal/mol |
| 1 | M_01 | ‒6.9 | 9 | M_111 | -8.6 |
| 2 | M_60 | ‒7.2 | 10 | M_112 | ‒7.7 |
| 3 | M_62 | ‒6.8 | 11 | M_201 | -7.9 |
| 4 | M_77 | ‒7.3 | 12 | M_366 | -8.0 |
| 5 | M_78 | ‒8.0 | 13 | M_421 | -9.3 |
| 6 | M_82 | ‒8.4 | 14 | M_505 | -8.9 |
| 7 | M_83 | ‒8.1 | 15 | Co-ligand N3 | -7.0 |
| 8 | M_88 | ‒8.1 |  |  |  |

| **Table S3**. Reported antiviral activities of mushroom compounds | | |
| --- | --- | --- |
| Name of compounds | Sources | Activity with reference |
| 1. Triterpines applanoxidic acid G, lucidadiol, and Ganodermadiol | Ganoderma species | Against influenza virus A and HSV 1 [1] |
| 2. Polysaccharide krestin (PSK) | *Trametesversicolor* (Turkey tail Mushroom) and *Lentinusedodes* mycelium | Extract inhibits cell to cell infection by HIV-1 and HIV-2  [2] |
| 3. Agrocybone | Agrocybesalicacola | Showed antiviral activity against respiratory syncytial virus (RSV) [3] |
| 4. Ganoderic acid, a triterpene from | *Ganodermalucidum* | Inhibits the replication of hepatitis B virus [4] |
| 5. Ganoderic acid A–C, ganoderic acid β, ganolucidic acid A, lucidumol B, ganodermanontriol, 3β-5α-dihydroxy-6β-methoxyergosta-7,22-diene, ganodermanondiol found in ganomycin I, colossolactones (A, E, G, V, VII, VIII), ganomycin-B from *G. colosum*; ganoderiol A, ganoderiol F, 20-hydroxylucidenic acid N, ganoderic acid GS-2, 20(21)-dehydrolucidenic acid N, lucidumol A | *Ganodermalucidum*; *G. sinnense* | Potential HIV-1protease inhibition activity at µM range [5, 6, 7, 8] |
| 6. Lactinsconcanavalin A | *Volvariellavolvacea* | activate T lymphocytes [9] |
| 7. Ricin-B-like lectin (CNL) | *Clitocybenebularis* | Stimulating dendritic cells (DCs) and cytokines. [9] |
| 8. Terpenoids, exobiopolymers | *Ganodermaapplanatum* | Activate Natural Killer (NK) cells, [9] |
| 9. Ganolucidoid A and B | *Ganodermalucidum* | Anti-inflamatory activity |
| 10. Lanostane | *Hypholomafasciculare* | Exhibit anti-inflamatory activity [9] |


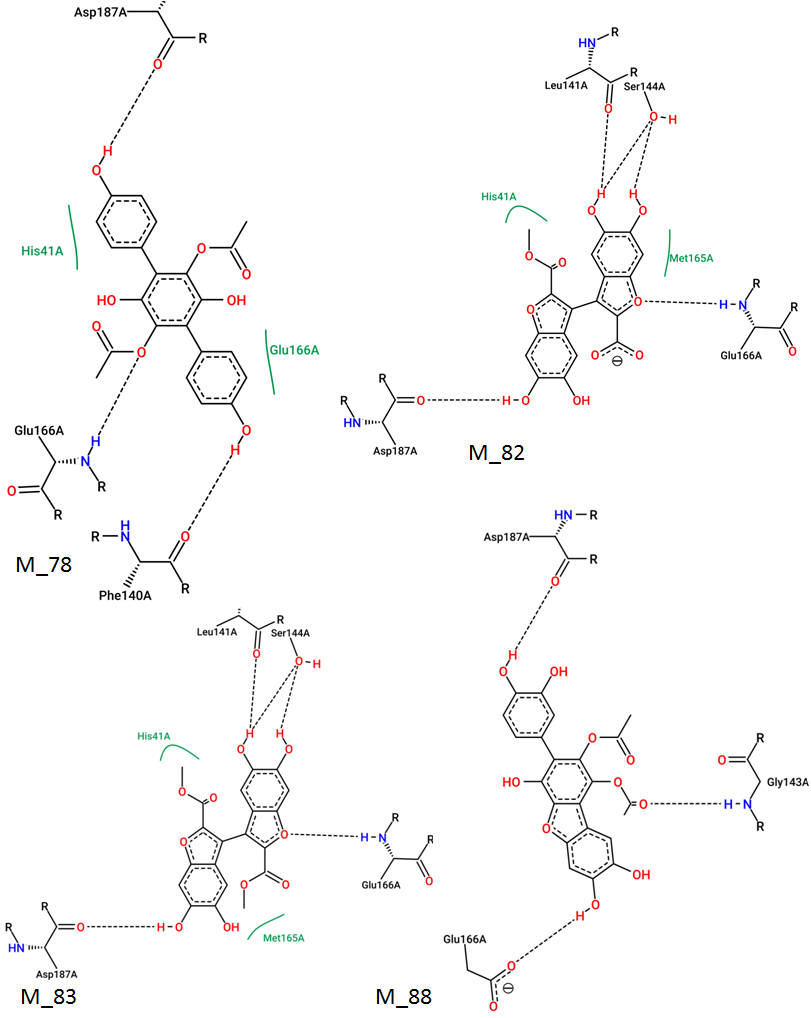


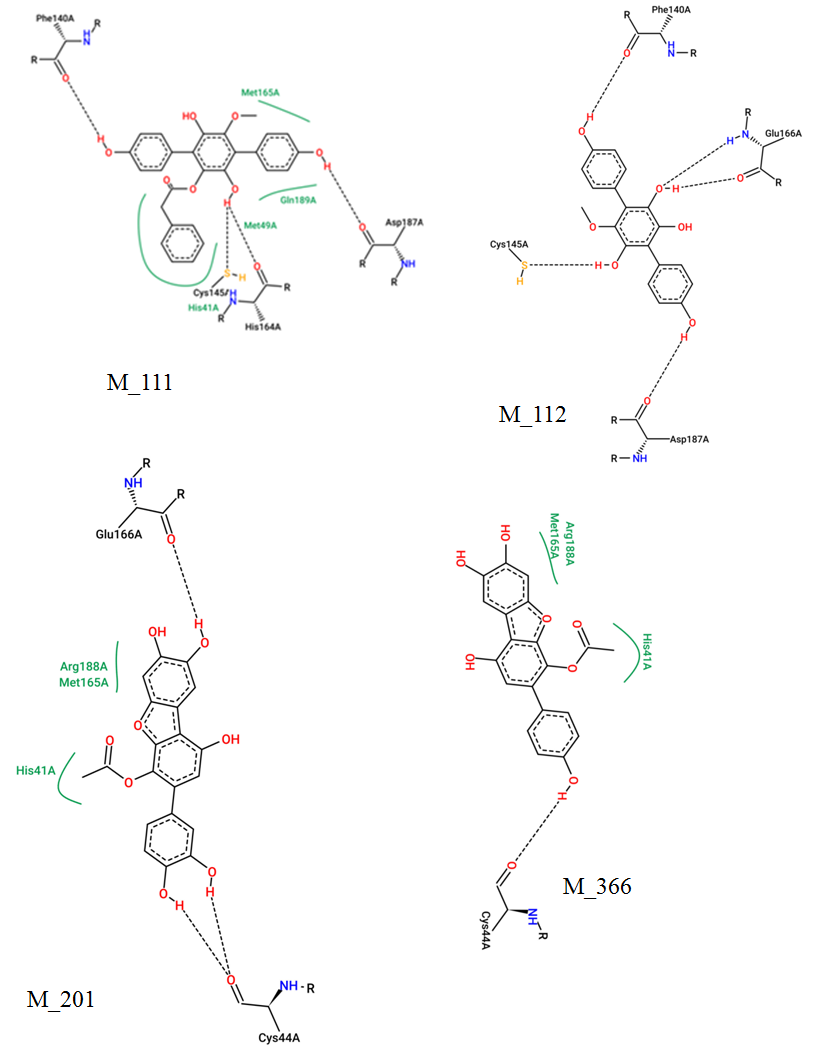


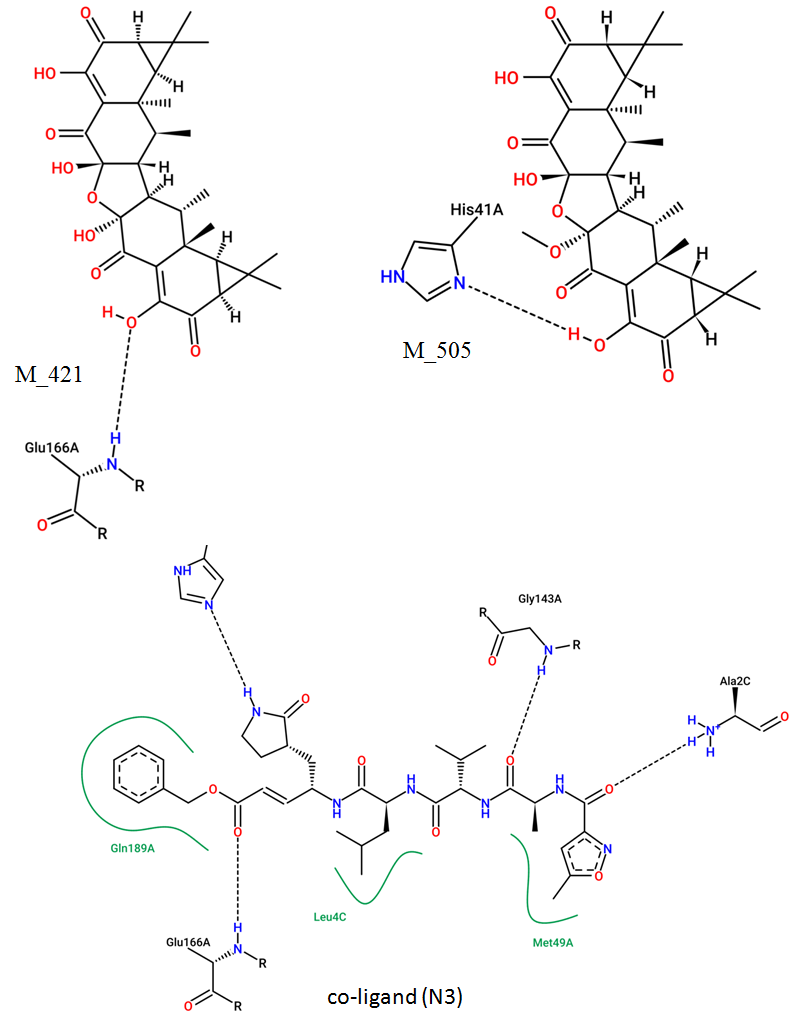


**Figure S1**. 2D ligand binding interaction analysis of selected hits (M_78, M_82, M_83, M_88, M_111, M_112, M_201, M_366, M_421, M_505 and coligand) with SARS-CoV-2 main protease (PDB ID: 6LU7). The dotted black line indicates H-bond donor-acceptor interaction, green colour residue indicates hydrophobic interactions.


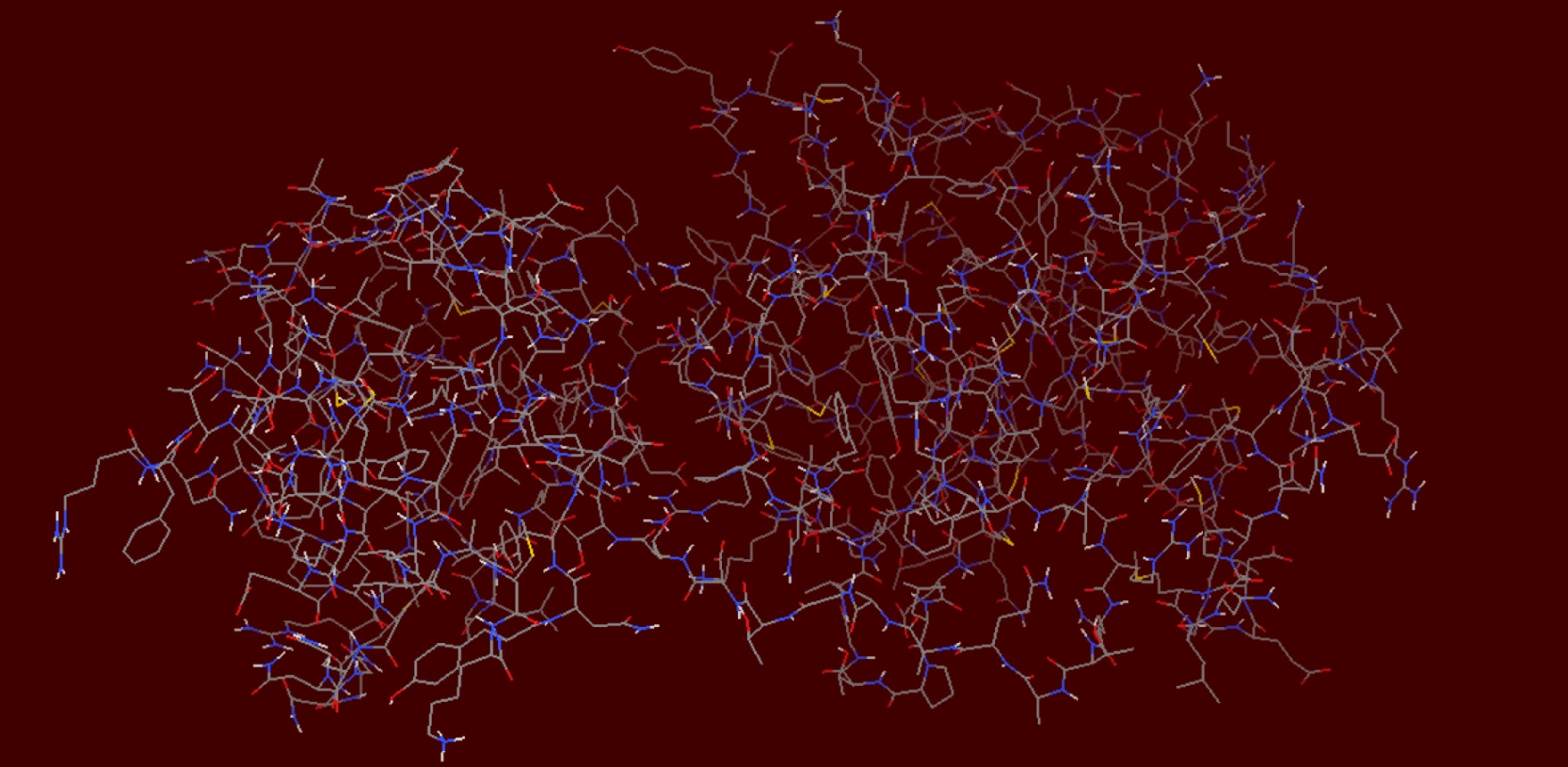


**Figure S2**. Protonated (pH 6-5) target protein (PDB ID: 6LU7) of SARS-CoV-2 M^pro^


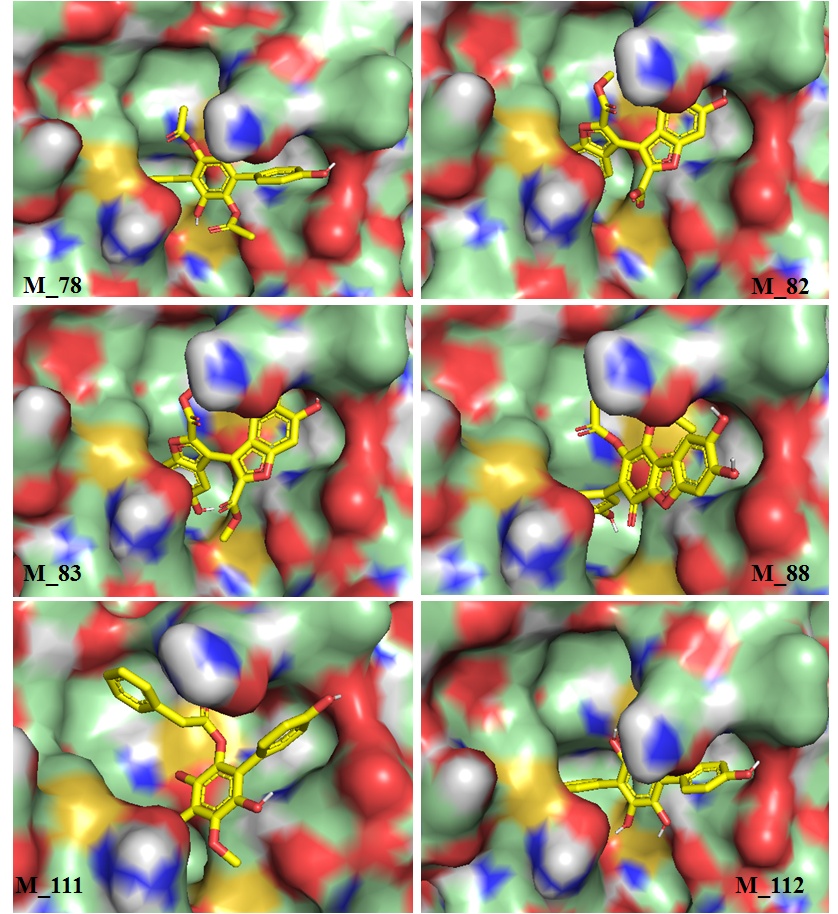


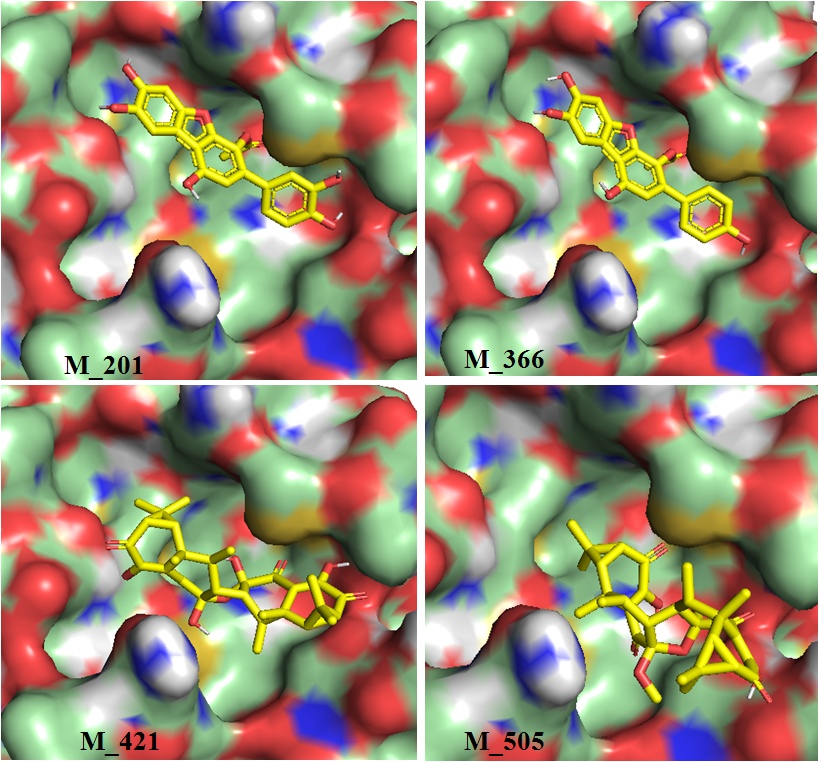


**Figure S3**. Binding pose in 3D space of selected hits (yellow) in the receptor-binding domain of SARS-CoV-2 M^pro^


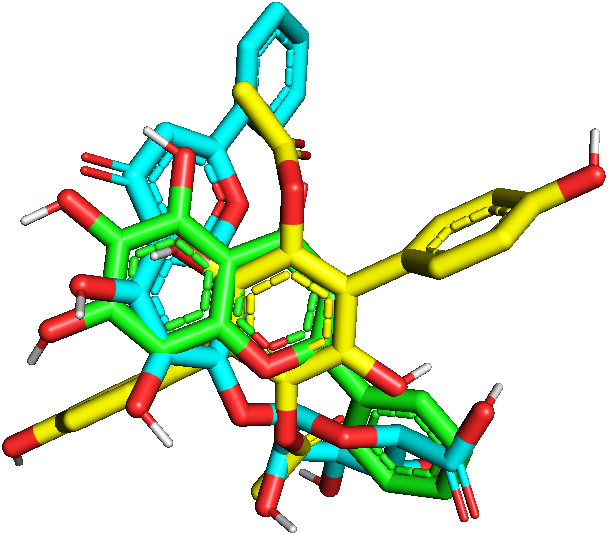


M_78 (yellow)-**1**-(green)-**2**-(sky) in the active site


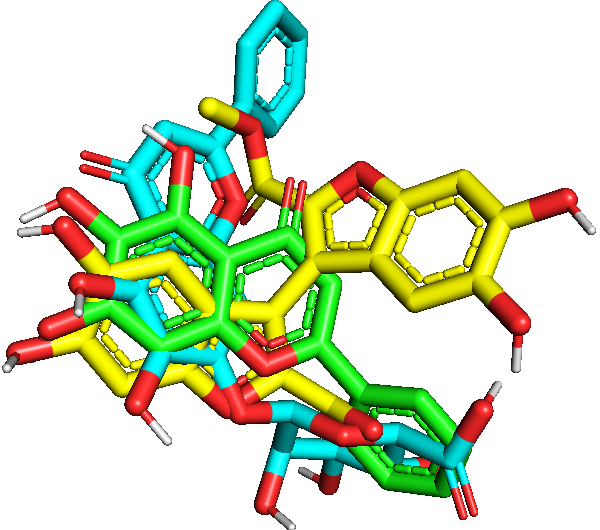


M_82 (yellow)-**1**-(green)-**2**-(sky) in the active site


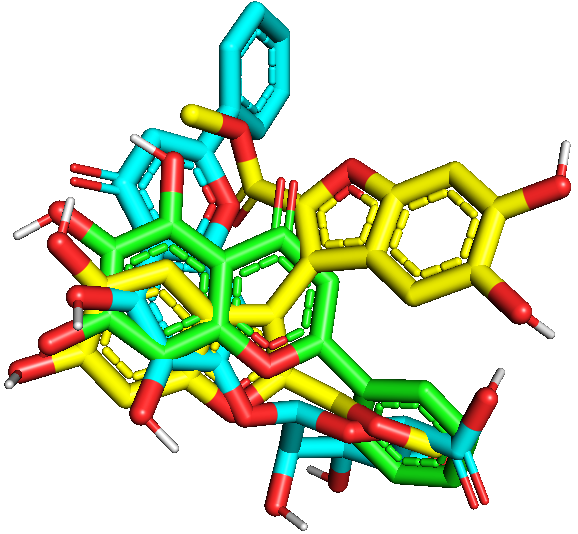


M_83 (yellow)-**1**-(green)-**2**-(sky) in the active site


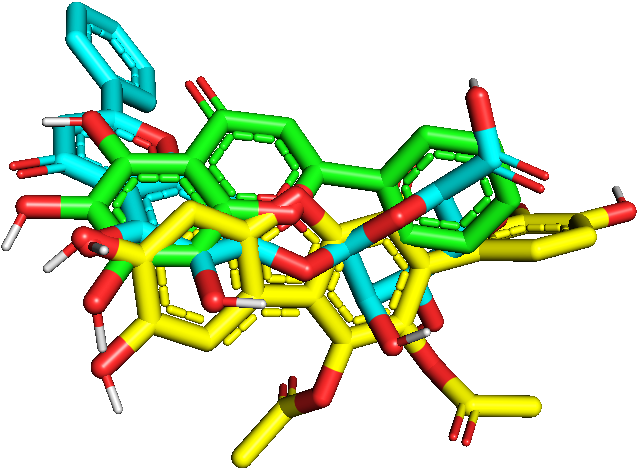


M_88 (yellow)-**1**-(green)-**2**-(sky) in the active site


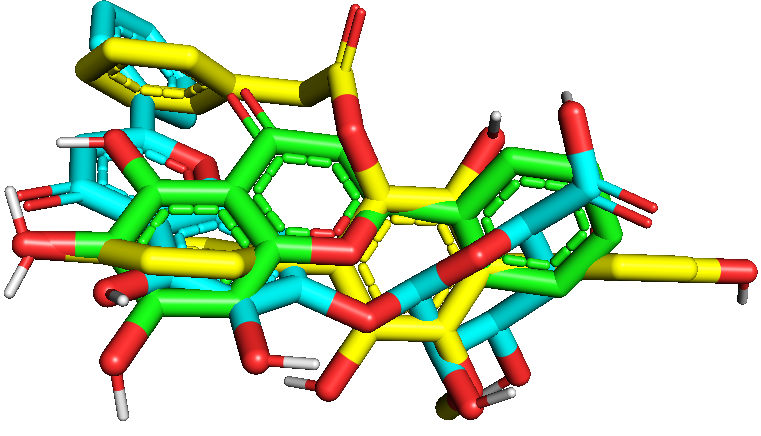


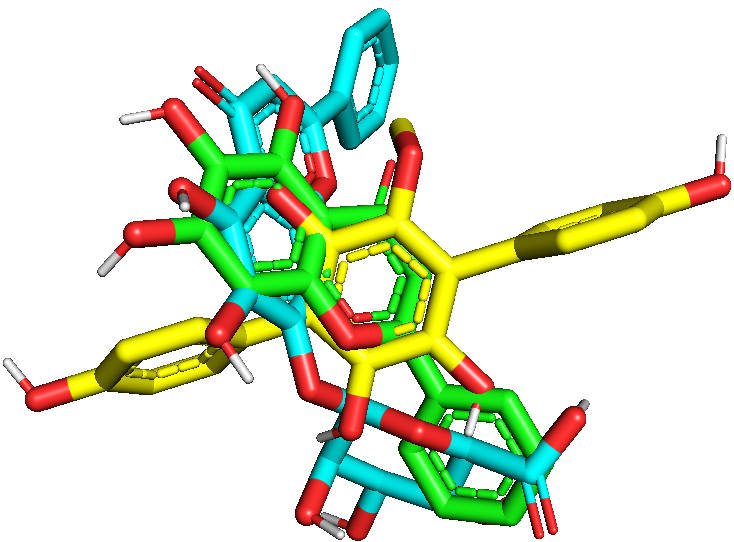
M_111 (yellow)-**1**-(green)-**2**-(sky) in the active site

M_112 (yellow)-**1**-(green)-**2**-(sky) in the active site


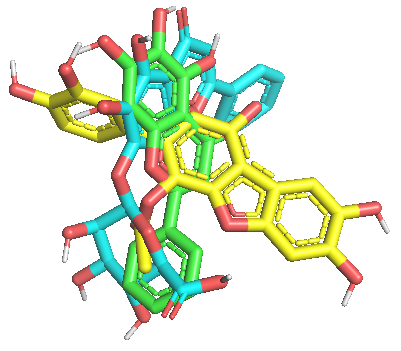


M_201 (yellow)-**1**-(green)-**2**-(sky) in the active site


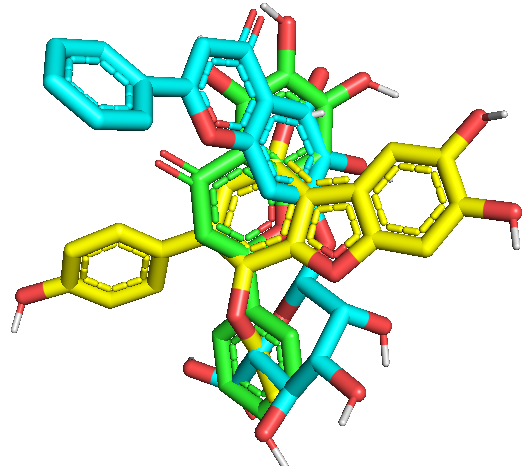


M_336 (yellow)-**1**-(green)-**2**-(sky) in the active site

**Figure S4**. Comparison of binding pose of selected hits (M_78, M_82, M_83, M_88, M_111, M_112, M_336) with known inhibitors **1**, **2** in the active site


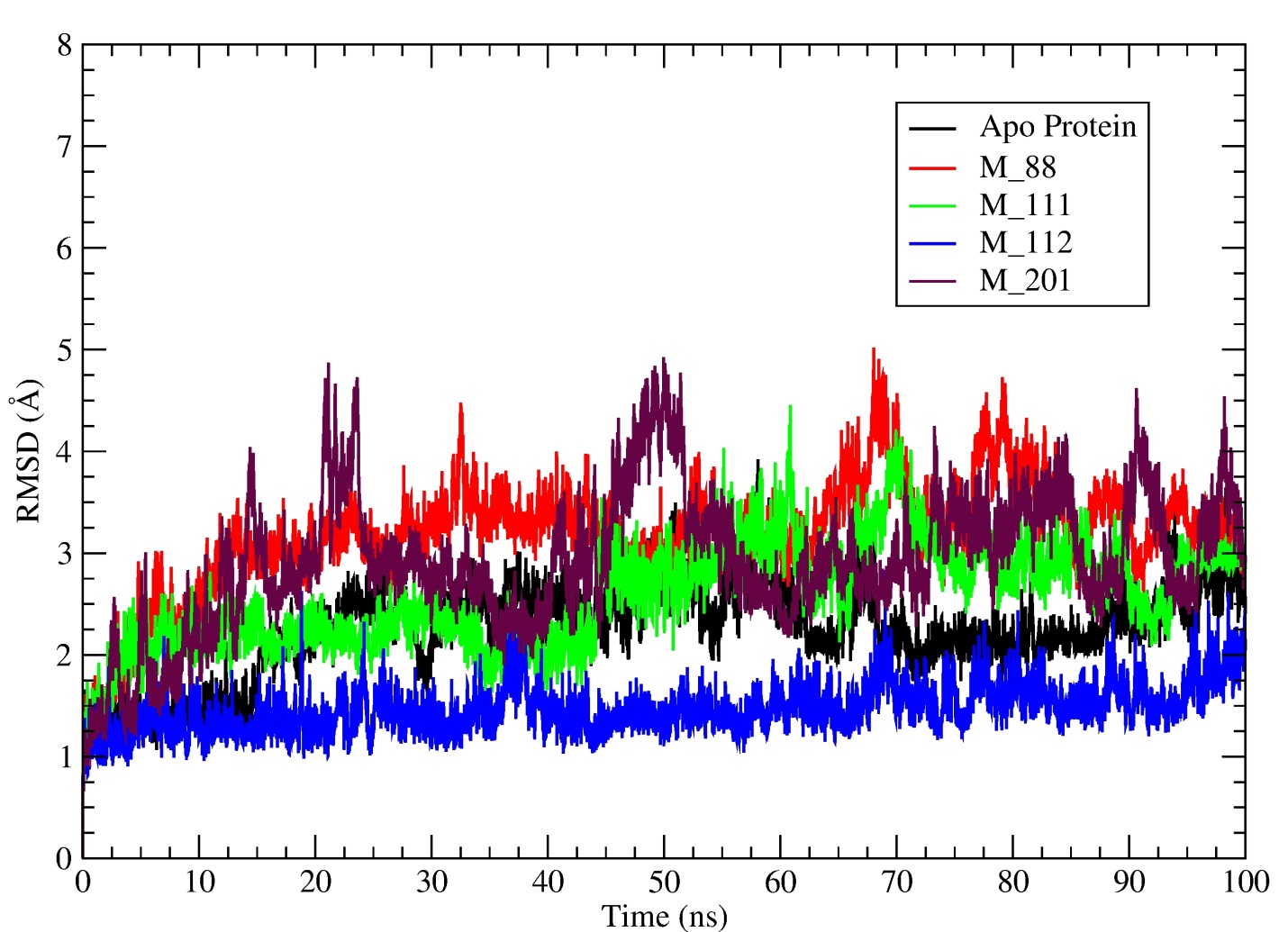


**Figure S5**. M^pro^ backbone RMSD of M^pro^‒ligand (M_88, M_111, M_112, M_201) complex and apo-protein obtained from 100 ns MD simulation trajectories


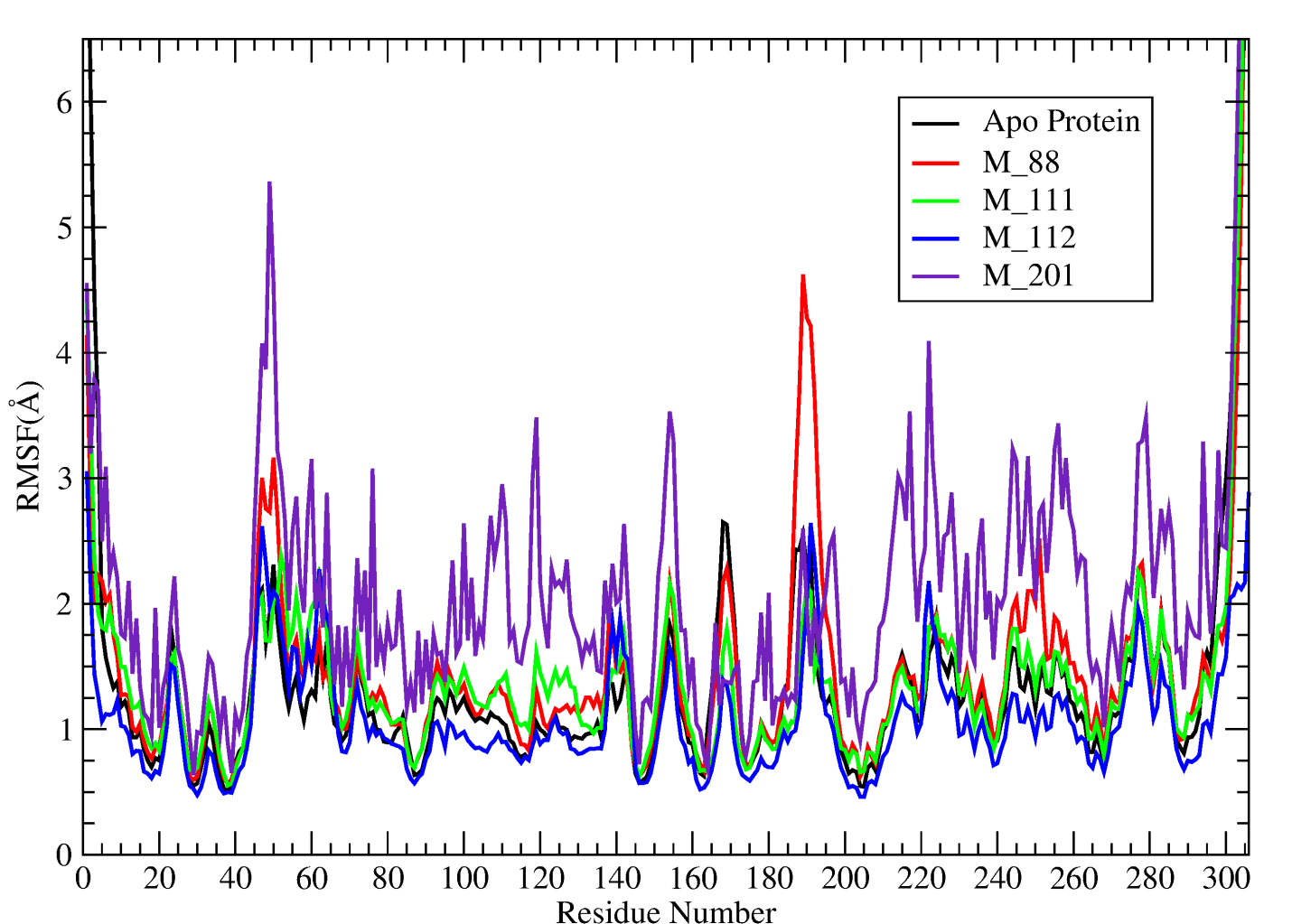


**Figure S6**. M^pro^ backbone RMSF vs residue number of M^pro^ –ligand (M_88, M_111, M_112, M_201) system and apo-protein during 100 ns simulation


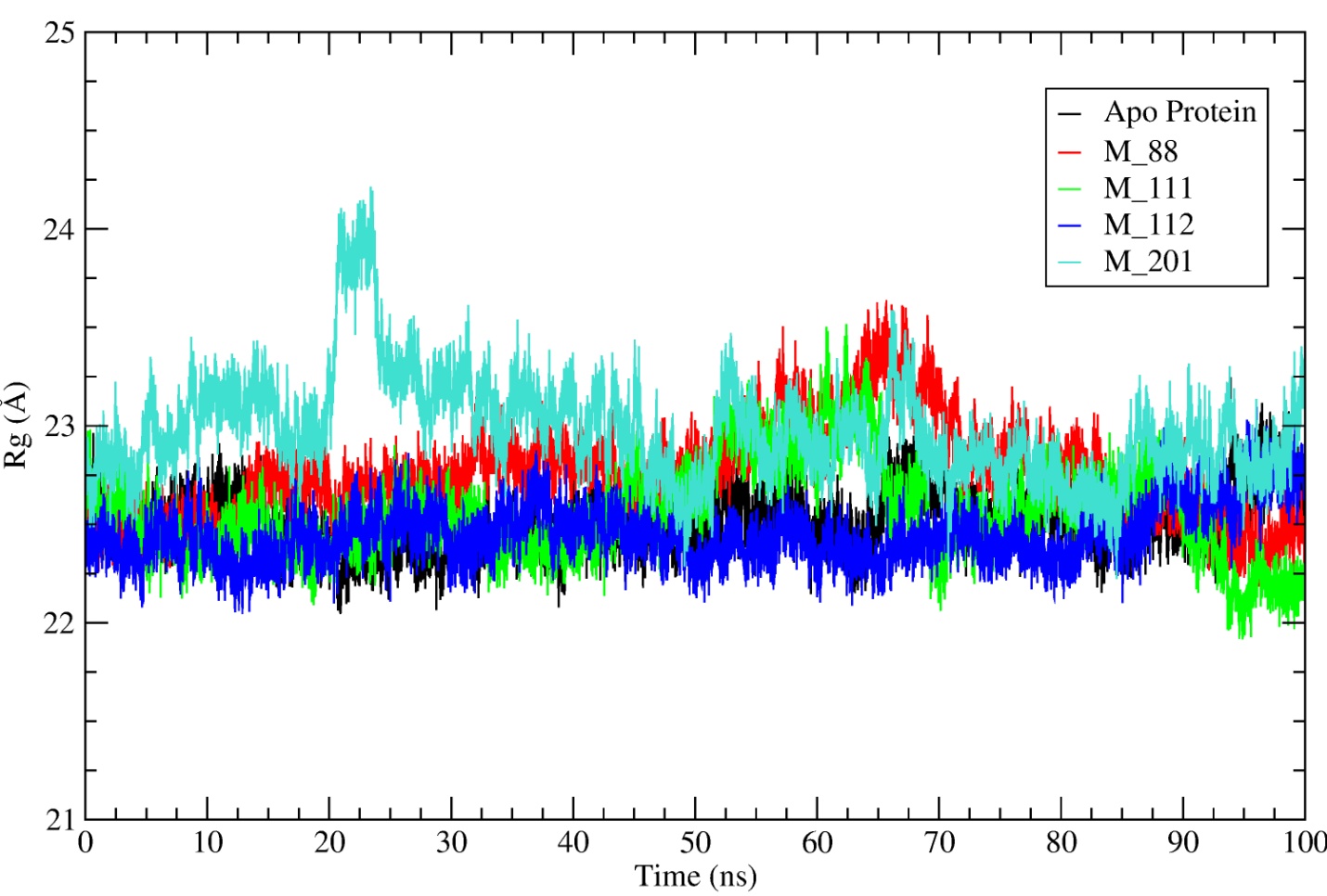


**Figure S7**. Radius of gyration vs time during 100 ns MD simulation of M^pro^‒ligand (M_88, M_111, M_112, M_201) systems and apo-protein


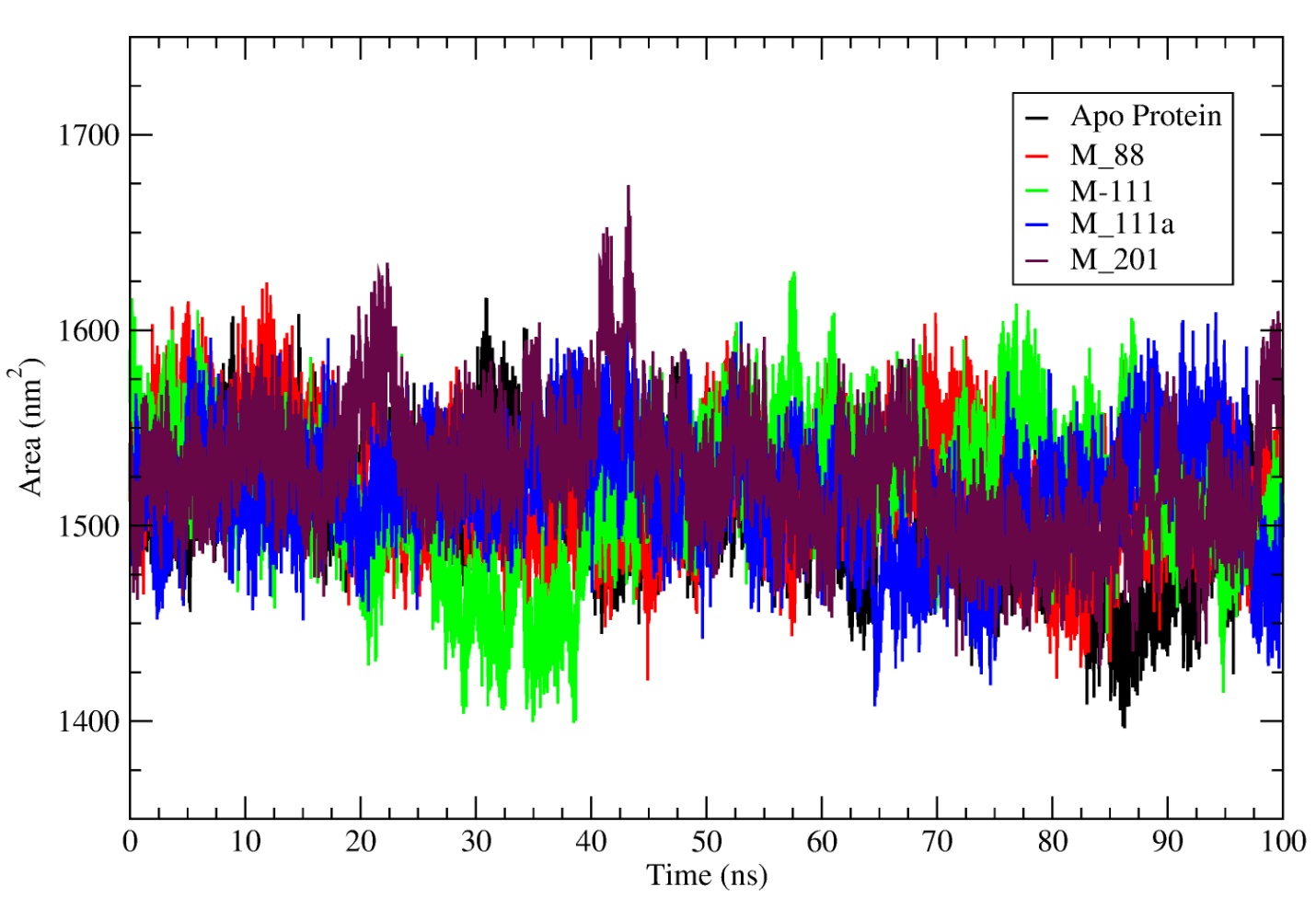


**Figure S8**. Solvent accessible surface area of M^pro^‒ligand (M_88, M_111, M_112, M_201) systems and apo-protein during 100 ns simulation time


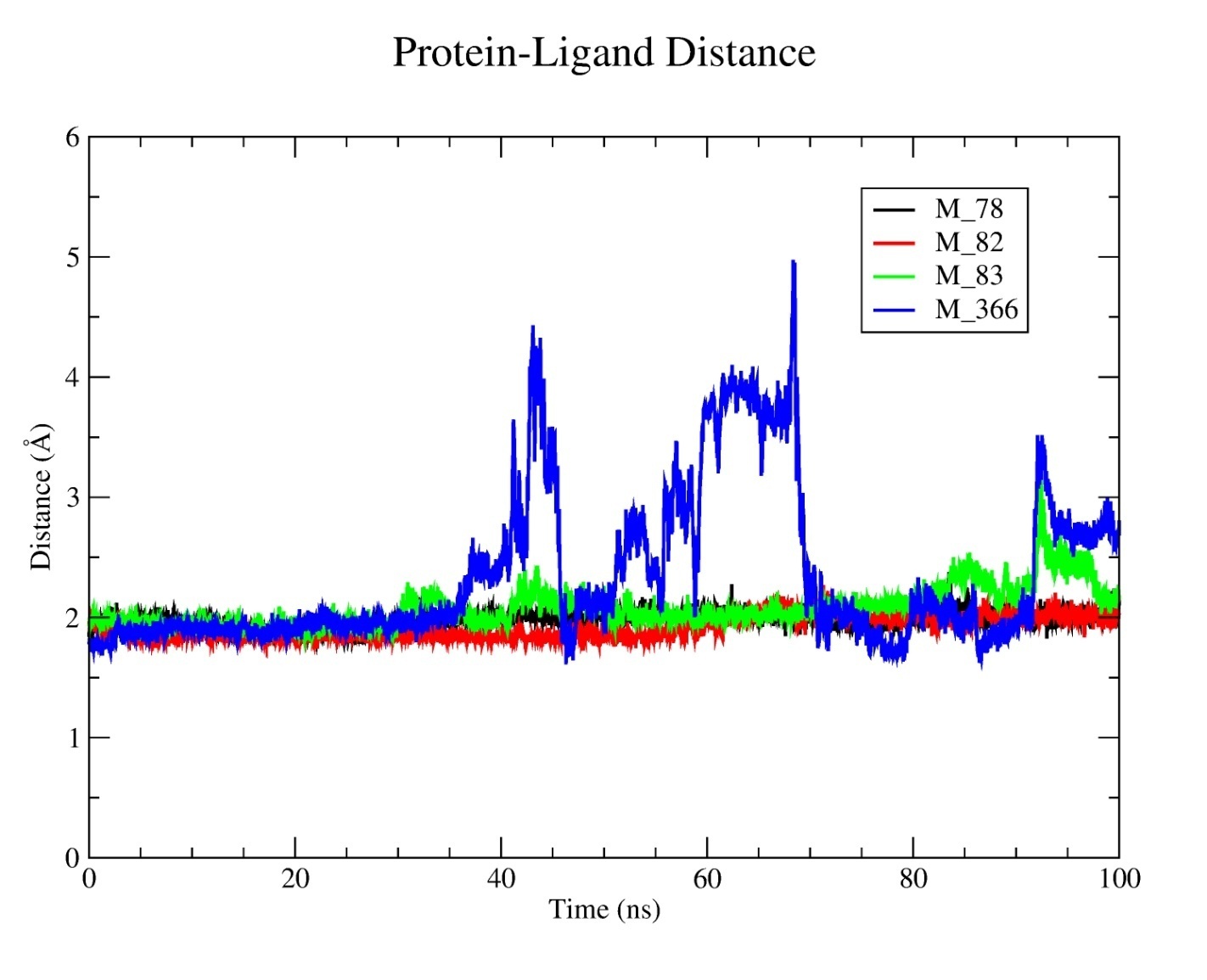


**Figure S9**. A visual inspection of the protein-ligand (78, 82, 83 and 366) distance i.e. center of mass (CoM) derived from 100 ns simulation time


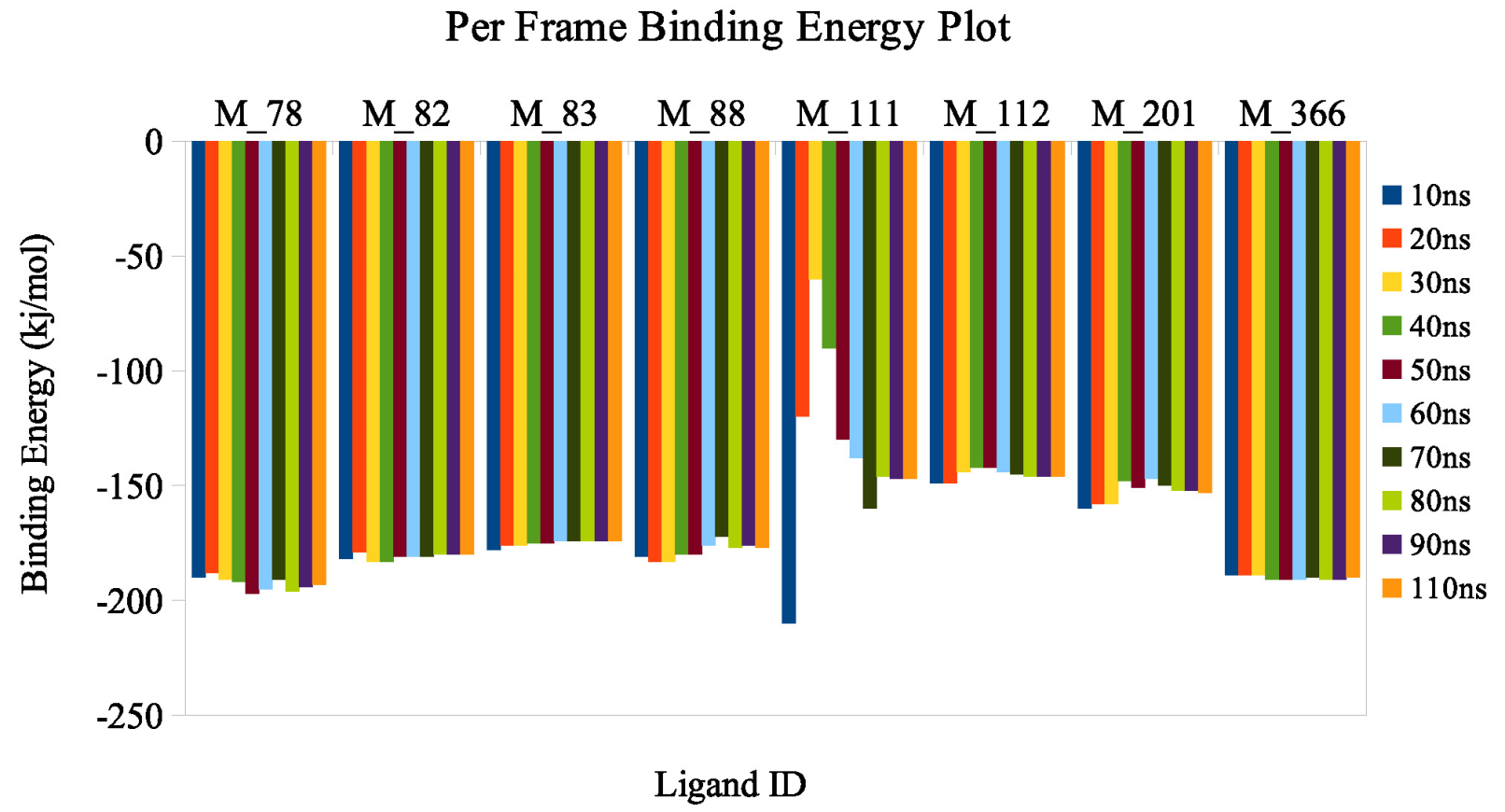


**Figure S10**. Visual inspection of per frame binding energy over the simulated time

**References**

1. Mothana RA, Awadh NA, Jansen R, Wegner U, Mentel R, Lindequist U.2003.Antiviral lanostanoidtriterpenes from the fungus *Ganodermapfeifferi* Bres. Fitoterapia. 74: 177–180

2. Tochikura TS, Nakashima H, Yamamoto N. 1989.Antiviral agents with activity against human retroviruses. *J Acquir Immune DeficSyndr (1988)*. 2(5):441-447

3. Zhu YC, Wang G, Yang XL, Luo DQ, Zhu QC, Peng T, Liu JK. 2010.Agrocybone, a novel bis-sesquiterpene with a spirodienone structure from basidiomyceteAgrocybesalicacola. TetrahedrLett. 51: 3443–5.

4. Li YQ, Wang SF. 2006. Anti-hepatitis B activities of ganoderic acid from *Ganodermalucidum*. *Biotechnollett*. *28(11)*, 837-841. <https://doi.org/10.1007/s10529-006-9007-9>

5. Min BS, Nakamura N, Miyashiro H, Bae KW, Hattori M. 1998. Triterpenes from the spores of *Ganodermalucidum* and their inhibitory activity against HIV-1 protease.*Chem. Pharm. Bull. 46(10)*, 1607–1612. https://doi.org/10.1248/cpb.46.1607

6. El Dine RS, Halawany AME, Ma CM, Hattori M. 2008. Anti-HIV1- protease activity of lanostanetriterpenes from the Vienamese mushroom *Ganodermacolossum*. *J. Nat. Prod*. *71(6)*, 1022–1026. https://doi.org/10.1021/np8001139

7. El Dine RS, El-Halawany A, Ma CM, Hattori,M. 2009.Inhibition of the dimerization and active site of HIV-1 protease by secondary metabolites from the Vietnamese Mushroom *Ganodermacolossum*. *J. Nat. Prod*. *72(11)*, 2019–2023. https://doi.org/10.1021/np900279u

8. Sato N, Zhang Q, Ma CM, Hattori M. 2009. Anti-human immunodeficiency virus-1 protease activity of new lanostane-type triterpenoids from *Ganodermasinense*. *Chem. Pharm. Bull*. *57(10)*, 1076–1080. <https://doi.org/10.1248/cpb.57.1076>

9. Suwannarach N, Kumla J, Sujarit K, Pattananandecha T, Saenjum C, Lumyong S. 2020. Natural Bioactive Compounds from Fungi as Potential Candidates for Protease Inhibitors and Immune modulators to Apply for Coronaviruses. *Molecules*. 25(8):1800. https://doi.org/10.3390/molecules25081800
